# Genome scans of facial features in East Africans and cross-population comparisons reveal novel associations

**DOI:** 10.1371/journal.pgen.1009695

**Published:** 2021-08-19

**Authors:** Chenxing Liu, Myoung Keun Lee, Sahin Naqvi, Hanne Hoskens, Dongjing Liu, Julie D. White, Karlijne Indencleef, Harold Matthews, Ryan J. Eller, Jiarui Li, Jaaved Mohammed, Tomek Swigut, Stephen Richmond, Mange Manyama, Benedikt Hallgrímsson, Richard A. Spritz, Eleanor Feingold, Mary L. Marazita, Joanna Wysocka, Susan Walsh, Mark D. Shriver, Peter Claes, Seth M. Weinberg, John R. Shaffer

**Affiliations:** 1 Department of Human Genetics, University of Pittsburgh, Pittsburgh, Pennsylvania, United States of America; 2 Department of Oral and Craniofacial Sciences, Center for Craniofacial and Dental Genetics, University of Pittsburgh, Pittsburgh, Pennsylvania, United States of America; 3 Department of Chemical and Systems Biology, Stanford University School of Medicine, Stanford, California, United States of America; 4 Department of Genetics, Stanford University School of Medicine, Stanford, California, United States of America; 5 Medical Imaging Research Center, Katholieke Universiteit Leuven, Leuven, Belgium; 6 Department of Human Genetics, Katholieke Universiteit Leuven, Leuven, Belgium; 7 Department of Anthropology, Pennsylvania State University, State College, Pennsylvania, United States of America; 8 Processing Speech & Images, Department of Electrical Engineering, Katholieke Universiteit Leuven, Leuven, Belgium; 9 Murdoch Children’s Research Institute, Melbourne, Victoria, Australia; 10 Department of Biology, Indiana University Purdue University Indianapolis, Indianapolis, Indiana, United States of America; 11 Department of Developmental Biology, Stanford University School of Medicine, Stanford, California, United States of America; 12 Applied Clinical Research and Public Health, School of Dentistry, Cardiff University, Cardiff, United Kingdom; 13 Anatomy in Radiology, Weill Cornell Medicine-Qatar, Doha, Qatar; 14 Department of Anatomy and Cell Biology, Alberta Children´s Hospital Research Institute, University of Calgary, Calgary, Canada; 15 Human Medical Genetics and Genomics Program, University of Colorado School of Medicine, Aurora, Colorado, United States of America; 16 Howard Hughes Medical Institute, Stanford University School of Medicine, Stanford, California, United States of America; 17 Department of Anthropology, University of Pittsburgh, Pittsburgh, Pennsylvania, United States of America; University of Pennsylvania, UNITED STATES

## Abstract

Facial morphology is highly variable, both within and among human populations, and a sizable portion of this variation is attributable to genetics. Previous genome scans have revealed more than 100 genetic loci associated with different aspects of normal-range facial variation. Most of these loci have been detected in Europeans, with few studies focusing on other ancestral groups. Consequently, the degree to which facial traits share a common genetic basis across diverse sets of humans remains largely unknown. We therefore investigated the genetic basis of facial morphology in an East African cohort. We applied an open-ended data-driven phenotyping approach to a sample of 2,595 3D facial images collected on Tanzanian children. This approach segments the face into hierarchically arranged, multivariate features that capture the shape variation after adjusting for age, sex, height, weight, facial size and population stratification. Genome scans of these multivariate shape phenotypes revealed significant (p < 2.5 × 10^−8^) signals at 20 loci, which were enriched for active chromatin elements in human cranial neural crest cells and embryonic craniofacial tissue, consistent with an early developmental origin of the facial variation. Two of these associations were in highly conserved regions showing craniofacial-specific enhancer activity during embryological development (5q31.1 and 12q21.31). Six of the 20 loci surpassed a stricter threshold accounting for multiple phenotypes with study-wide significance (p < 6.25 × 10^−10^). Cross-population comparisons indicated 10 association signals were shared with Europeans (seven sharing the same associated SNP), and facilitated fine-mapping of causal variants at previously reported loci. Taken together, these results may point to both shared and population-specific components to the genetic architecture of facial variation.

## Author summary

Genetic factors play an important role in shaping human facial features. Over the last decade, studies have identified numerous genes associated with various facial traits. The vast majority of these studies have focused on European or Asian populations, while African populations have been underrepresented. Increasing the diversity of these analyses can reveal novel associations and cross-population analyses can help deepen our understanding of known genetic associations. We therefore performed a genome scan of 3D facial features in African children from Tanzania and then compared our results to Europeans. We found 20 regions of the genome associated with facial shape in Tanzanian children, 10 of which were also present in Europeans, indicating evidence for a partly shared genetic basis for human facial shape across populations. In addition, about half of the genetic associations observed in Tanzanians were not present in Europeans, and some of the shared signals differed between populations in the specific genetic variants associated or specific facial traits affected. These results shed light on the shared and population-specific genetic contributors to normal-range facial variation.

## Introduction

The human face shows a wide range of variation in shape. Although facial features change across the lifespan and can be influenced by environmental factors such as nutritional status, numerous lines of evidence from twin and family studies show that the majority of variation in facial shape is determined by genetics, with the narrow-sense heritability of facial traits estimated to be approximately 40% to 60% [1–3]. To date, at least 17 genome-wide association studies (GWAS) of facial traits have been performed, including 11 in European [3–13], two in Asian [14–16], one in Latin American [17], one in African [18], and two in mixed-ancestry populations [19,20]. These studies have used varied phenotyping strategies, and some have been quite successful in identifying genetic variants associated with aspects of facial morphology. For example, our recent GWAS meta-analysis of data-driven phenotypes derived from 3D images reported 203 signals across 138 genetic loci showing genetic associations with facial traits in Europeans [13]. In contrast, far fewer loci have been identified in non-European populations, and even fewer have been replicated across populations. Indeed, only eight have shown genome-wide significant associations across different ancestral groups (HOXD cluster, *PAX3*, *TBX3*, *SOX9*, *PAX1*, 4q31.3, 6p21.1, 20q12). African populations are particularly under-represented in facial GWA studies. The only previous GWAS of facial morphology in an African population was performed by Cole et al. using landmark-based phenotypes extracted from 3D facial images in 3,505 Bantu children and Mwanza adolescents [18]. This study did not replicate any previously identified loci, but did report two genetic associations, the *SCHIP1* locus with centroid size and the *PDE8A* locus with the allometric variation in facial shape. Associations with these two loci have not been reported in other populations.

While this lack of overlap among associated loci across populations may be attributable to differences across studies in phenotyping modalities or insufficient power to detect small effects, it may also reflect true differences in the genetic architecture across populations. Few studies have explicitly sought to explore this question, and consequently, the degree to which facial traits share a common genetic basis across diverse populations remains largely unknown. Genome-wide scans of facial variation have investigated varied phenotypes, typically using traditional anthropometric landmarks as the basis for deriving phenotypes. However, genetic associations discovered using such approaches have been limited, likely due to the inadequacy of the simple landmark-based phenotypes in capturing the complex morphology of the face. Therefore, we previously developed a global-to-local phenotyping approach that allowed us to more fully utilize the integrated information captured from 3D facial images. This method has been applied to GWASs of European ancestry samples with great success [10,13]. In the present study, we applied the global-to-local phenotyping approach to a previously collected East African sample reported by Cole et al. [18]. We performed GWAS of facial morphology in 2,595 East Africans and compared results with those from an independent GWAS of 8,246 European-ancestry participants. Our re-analysis of this dataset points to both shared and possible population-specific associations, which deepen our understanding of the genetic architecture of normal facial variation and provides insights into the genetic underpinnings of craniofacial dysmorphology and the embryonic origin of facial morphogenesis.

## Methods

### Ethics statement

Tanzania discovery cohort: Written informed consent was obtained from all Tanzanian study participants or their parents as appropriate. Ethics approval for the overall study was obtained at the University of Colorado (protocol #09–0731), with additional institutional approvals at the University of Calgary, and the Catholic University of Health and Allied Sciences (Mwanza, Tanzania) in conjunction with the Tanzania National Institute of Medical Research. European replication cohorts: Institutional review board approval was obtained at each recruitment site. For the US-based cohorts this approval included the University of Pittsburgh (PITT IRB PRO09060553 and RB0405013), Seattle Children’s Hospital (Seattle Children’s IRB 12107), University of Texas Health Science Center at Houston (UT Health Committee for the Protection of Human Subjects HSC-DB-09-0508), University of Iowa (University of Iowa Human Subjects Office IRB 200912764 and 200710721), the Pennsylvania State University (PSU IRB #’s 13103, 45727, 2015–3073, 2503, 44929, 4320, 44929, and 1278), and Indiana University (IUPUI IRB 1409306349). For the UK-based cohort, ethical approval was obtained from the Avon Longitudinal Study of Parents and their Children (ALSPAC) Ethics and Law Committee and the Local Research Ethics Committees. Informed consent for the use of data collected via questionnaires and clinics was obtained from participants following the recommendations of the ALSPAC Ethics and Law Committee at the time. Consent for biological samples has been collected in accordance with the Human Tissue Act (2004). Written informed consent was obtained from all participants or their parents before participation.

### Recruitment and data collection

This study is a re-analysis of the dataset described by Cole et al [18]. The African cohort included 3,555 participants from the Mwanza region of Tanzania comprising 1,582 males and 1,973 females aged 3 to 21 years (S1 Fig). 3D facial images were collected using the Creaform MegaCapturor (MC) camera three-dimensional (3D) photogrammetric imaging system or the Creaform Gemini (GM) 3D imaging system. 3D facial images were obtained while participants maintained closed mouths and neutral, relaxed facial expressions during image capture. Exclusion criteria included personal history of a known birth defect or family history of an orofacial cleft. The sample included some related participants; therefore, one member of each kinship was randomly chosen for inclusion. A total of 960 participants were excluded from analysis based on exclusion criteria, resulting in 2,595 unrelated participants that were retained for the genetic analysis. Population structure of the Tanzania cohort was assessed using principal component analysis (PCA) of genotyped SNPs chosen for high call rate (>95%), minor allele frequency (MAF) >0.05 and low linkage disequilibrium (LD; pairwise r^2^ <0.1 across variants in a sliding window of 10 Mb). Based on the scree plot and joint distributions, we determined that four principal components (PCs) were sufficient to adjust for the effect of population structure within the sample [18] (see S2 Fig).

Genotyping for the Tanzania cohort was performed using the Illumina HumanOmni2.5Exome-8v1_A array by the Center for Inherited Disease Research (CIDR) of Johns Hopkins University. Quality control procedures were performed to exclude low-quality single nucleotide polymorphisms (SNPs) and samples, as described previously [18]. Imputation was performed using the 1000 Genomes Project reference. Filters for imputation INFO score <0.8, genotype-per-participant probability <0.9, missing imputation rate <0.5, MAF <0.01, and deviations from Hardy-Weinberg equilibrium (p value < 1 × 10^−6^) were used to exclude SNPs from analysis. In total, >1.5M SNPs were included in the GWAS.

### Phenotyping

Phenotyping was performed using the pipeline described in Claes *et al*. [10]. The 3D surface images were imported into Matlab in wavefront.obj format, and processed using the “MeshMonk” open-source package [21]. First, the individual 3D images were cropped and trimmed to remove hair and imaging artifacts. Five landmarks were placed on each face in a consensus reference frame, which established a rough image orientation. A bilateral symmetrical anthropometric mask of 7,160 quasi-landmarks was subsequently mapped onto the 3D images. A Generalized Procrustes Analysis (GPA) was used to eliminate the differences in the orientation, position, and size of the quasi-landmark configurations. This work focused on the symmetrical variation in facial phenotypes by averaging quasi-landmark positions between left and right sides of the face of images with their reflections.

Quality control was performed on facial images to identify outliers that were likely due to image mapping errors. First, outlier faces were identified by measuring the Mahalanobis distance transformed to a z-score. Images with z-scores of >2, indicative of atypical facial shape, were visually inspected. Second, a metric was calculated to gauge image artifacts such as holes and spikes, which indicates missing parts or errors during processing steps. Images with high scores were visually inspected. After visual inspection, outlier images and images with artifacts were either excluded due to poor quality or re-mapped.

To generate facial shape phenotypes for genetic analysis, we performed a global-to-local facial segmentation process. Facial shape was first adjusted for covariates (including age, sex, height, weight, facial size [centroid-size], and genomic principal components), and then hierarchically partitioned into facial segments using an unsupervised and data-derived strategy [10]. This phenotyping method resulted in 63 partially overlapping facial segments arranged across five levels in a bifurcated hierarchical manner (S3A Fig). After the global-to-local segmentation step, each of the 63 facial segments was subjected to another GPA followed by a Principal Component Analysis (PCA) across the 3D coordinates of the quasi-landmarks within the segment for dimensionality reduction. Parallel analysis was used to determine the number of PCs retained, resulting in sets of PCs (6 to 57) capturing most of the shape variation (95% to 98%) in each facial segment [22].

### GWAS

The genetic association between each SNP and variation in each of the 63 facial segments (each represented by a set of PCs) was tested using canonical correlation analysis (CCA) under the additive genetic model as implemented in the “canoncorr” function in Matlab. This resulted in the linear combination of PCs that maximized the correlation with the SNP. Since the CCA approach cannot incorporate the effects of covariates, adjustments for sex, age, age-squared, height, weight, facial size, and four principal components of ancestry were made prior to testing, as previously described [10]. Significance of the CCA was determined by Rao’s F-test approximation (right tail, one-sided test). Associations with p-values < 2.5 × 10^−8^, the genome-wide significance threshold for African populations, were annotated [23]. The 63 facial segments represent partially overlapping regions of the face; therefore, the effective number of independent phenotypes tested was determined to be 40 based on the method by Li and Ji [24]. A study-wide significance threshold was set at p-value < 6.25 × 10^−10^ (i.e., 2.5 × 10^−8^/40) to account for the multiple testing burden due to the multiple, partially overlapping, facial segments.

### Gene annotation

We utilized the Ensembl Biomart toolset to identify the genes located within a 500Kb window (250Kb downstream and upstream) of lead GWAS SNPs. We searched the literature for evidence of the involvement of nearby genes in craniofacial development, morphology, or dysmorphology. Based on this corroborating evidence, the potential candidate genes for each leading SNP were noted.

### Expression quantitative trait locus (eQTL) co-localization analysis

For each genetic locus identified in the Tanzania cohort, we extracted the summary statistics of SNPs within 500Kb up- or downstream of lead SNP from the GWAS results and downloaded their eQTL data from the Genotype-Tissue Expression (GTEx) project (version 7). The “locuscompare” function in R program v3.6.1 was used to estimated co-localization of facial-associated variations and eQTLs using six tissues relevant to craniofacial morphology (i.e. adipose subcutaneous, adipose visceral omentum, fibroblasts, muscle skeletal and two skin tissues) [25].

### Cell-type-specific enhancer enrichment

Enhancer enrichment analyses were performed as described in our previous study [13]. In brief, fastq-format Chip-seq data of histone H3 on lysine K27 (H3K27ac) signal were downloaded from the University of California, Santa Cruz (UCSC) Genome Browser and Gene Expression Omnibus (GEO) [26–30]. The tagAlign-format Chip-seq data of H3K27ac signal (GSE; embryonic craniofacial tissue) [31] and the Roadmap Epigenomics Project (https://egg2.wustl.edu/roadmap/data/byFileType/alignments/consolidated/; various fetal and adult tissues and cell-types) [32] were downloaded. Chromosomal coordinates of both Chip-seq data types were aligned to the human genome build GRC37/hg19. We divided the genome into 20 kb windows and used bedtools coverage (v2.27.1) to calculate H3K27ac reads per million (RPM) from each of the aligned read files in each window. We then normalized the matrix of 154,614 windows and 133 ChIP-seq data sets using the “normalize.quantiles” function in R program. The windows containing the lead SNP of each genome-wide significant locus were used for enrichment analysis.

### In-silico replication of Tanzanian hits in a European dataset

Results from the Tanzania discovery sample were compared to an existing meta-GWAS of European ancestry. The European cohort was comprised of 8,246 participants, including a combination of three datasets from United States (US) and a dataset from United Kingdom (UK). The UK dataset included samples from the ALSPAC study [33,34], a longitudinal birth cohort in which pregnant women residing in Avon, UK with an expected delivery date from 1st April 1991 to 31st December 1992 were recruited. At the time, 14,541 pregnant women were recruited and DNA samples were collected for 11,343 children. Genome-wide data was available for 8,952 subjects of the B2261 study, titled “Exploring distinctive facial features and their association with known candidate variants.” The intersection of unrelated participants of European ancestry with quality-controlled images, covariates, and genotype data included 3,566 individuals. The ALSPAC study website contains details of all the data that is available through a fully searchable data dictionary and variable search tool (http://www.bris.ac.uk/alspac/researchers/our-data/). Details of three US datasets, including the study enrollment, collection of high-resolution 3D facial images, phenotyping, genotyping, and genetic analysis, have been described previously [13]. S4 Fig presents the workflow of the study, including the data collection of Tanzania and European-ancestry cohorts.

Direct replication testing was complicated by two features of the study design. First, the data-driven facial segmentation process and PCA of the quasi-landmarks used to generate the multivariate phenotypes are specific to each dataset. That is, the 63 segments are not exactly comparable across studies (S3 Fig). Second, the linear combination of PCs identified in the CCA analysis is specific to each SNP and each dataset. Therefore, the SNP associations reported in the Tanzania cohort do not necessarily represent the same morphological variation as in the European meta-GWAS. To address these issues, we performed five types of *in silico* replication analyses in the European meta-GWAS: (Test 1 [T1]) SNP-level testing (via linear regression) of the projection of the European-ancestry dataset onto the Tanzania derived phenotype (e.g. facial segmentation, PCs, and linear combination of PCs defined in the CCA) as a univariate phenotype; (T2) SNP-level look-up for the "best segment" (i.e., the segment showing the most significant evidence of association across the 63 segments); (T3) locus-level (+/- 500kb from lead SNP) look-ups for the "best segment"; (T4) SNP-level look-up for a qualitatively similar facial segment; (T5) locus-level look-ups for a qualitatively similar facial segment. The statistical analysis was implemented separately for each combination of genome-wide significant SNPs and corresponding facial segments.

The projected phenotype approach (T1) ensures that traits being compared across the two cohorts are equivalent and provides a means of directly replicating in Europeans the same genotype-phenotype relationship identified in the Tanzanian GWAS. The SNP-level look-ups in qualitatively similar and “best” segments allows the effect of a variant to differ across African and European ancestry groups. The locus-level look-ups allows for genetic differences (e.g., in linkage disequilibrium patterns, minor allele frequencies, allelic heterogeneity) across African and European ancestry groups. Cumulatively, these approaches (T1-T5) allow for detection of effects that meet different criteria for inter-ethnic replication. The projected phenotype approach (T1) provides the most direct means of replicating the same genotype-phenotype relationship identified in the Tanzanian GWAS, whereas the similar segment approach (T4) relaxes the need for the facial effect to be exactly identical. However, both of these approaches could miss replicating effects due to differences in LD structure across populations if the tested SNP is not actually causal. The locus-level approaches (T3 and T5) can accommodate detection of different causal variants, or different LD-based proxies of the same unobserved causal variant, across populations. The “best segment” approaches (T2 and T3) can accommodate detection of the associated variants that manifest differently across populations given the distinct facial morphologies in Europeans and Africans.

For replication tests (T1—T5), the significance threshold after Bonferroni correction was determined as 0.05 divided by the number of GWAS signals tested for replication. For the best segment SNP/locus-level replication test (T2 and T3), this threshold was further divided by the number of independent facial segments (n = 40). For the locus-level tests (T3 and T5), we further divided by the effective number of SNPs [24] at the locus or used the genome-wide association threshold of 5 × 10^−8^ in the European population, whichever was greater, as the significant threshold for replication.

For loci showing genome-wide association in the Tanzania cohort, co-localization analysis was performed based on the association between the best facial segment in the Tanzania cohort and a comparable segment in the European sample. Co-localization analysis was performed using the “locuscompare” function in R.

### In silico replication of European hits in the Tanzania dataset

The previous study in Europeans reported 203 significant associations with facial variation. To explore these associations in the Tanzania cohort, we performed three *in silico* replication tests (analogous to those previously described for replicating Tanzanian associations in Europeans): (T1) SNP-level testing of the projection of the Tanzania dataset onto the European derived phenotype; (T2) SNP-level look-up for the "best segment" and (T3) locus-level look-ups for the "best segment". The significance threshold after Bonferroni correction was determined in the same way as described in the replication analyses in the European meta-GWAS.

### In silico replication of previously reported landmark-based and qualitative trait associations in the Tanzania dataset

In addition to the previous studies in Europeans using the same data-driven global-to-local phenotyping approach as used here, there have been 14 GWAS and one whole-exome sequencing study, to date, using *a priori* landmark-based (e.g., linear distances, ratios, etc.) and qualitative phenotypes (e.g., self-reported chin dimples, etc.). The associated variants from these studies are summarized in S1 Table, including a total of 112 loci that have been implicated in previous studies at the genome-wide threshold for significance. We then investigated these 112 GWAS signals for their association with facial variation in the Tanzania cohort by conducting two *in silico* replication tests (T2 and T3) using the same methods as described above for determining the significant threshold after Bonferroni correction.

## Results

### Facial segmentation

**Fig 1:**
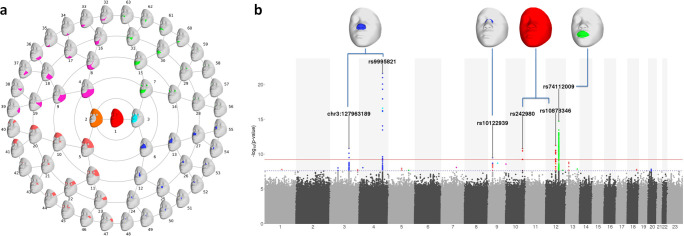
Facial segmentation and GWAS results. (a) Rosette showing the global-to-local partitioning of the full face into segments. The full face (segment 1, red) is first partitioned into segments representing the outer (2, orange) and inner (3, cyan) regions of the face. These are in turn partitioned into more localized regions representing the lower face (magenta), upper face (salmon), nose (blue), and mouth and eyes (green). (b) Combined Manhattan plot highlighting the genome-wide significant genetic variants across 63 facial segments. Significantly associated variants are colored to correspond to the facial segments as shown in (a). The blue dotted line and red solid line indicate the genome-wide (P < 2.5 × 10^−8^) and study-side (P < 6.25 × 10^−10^) significance thresholds, respectively.

The data-driven global-to-local phenotyping procedure yielded 63 hierarchically arranged facial segments, as shown in Fig 1A. The whole face was first split into two regions representing the midface (segment 3) and the outer face (segment 2), and then further partitioned into regions representing the lower face (quadrant 1), the mouth and regions around the eyes (quadrant 2), the nose (quadrant 3), and the upper face (quadrant 4). Variation in each segment was represented by 6 to 57 PCs, with the more global segments generally requiring more PCs to capture the variation than the more local segments (S2 Table). The facial segmentation was similar to that in a previous study of Europeans [10,13], with both yielding quadrants that represent variation in lips, nose, upper face and lower face area (S3 Fig). However, the Tanzania sample yielded a distinct sub-quadrant representing shape variation in the eye area, which was absent in the European-driven map.

### GWAS results in the Tanzania cohort

**Fig 2:**
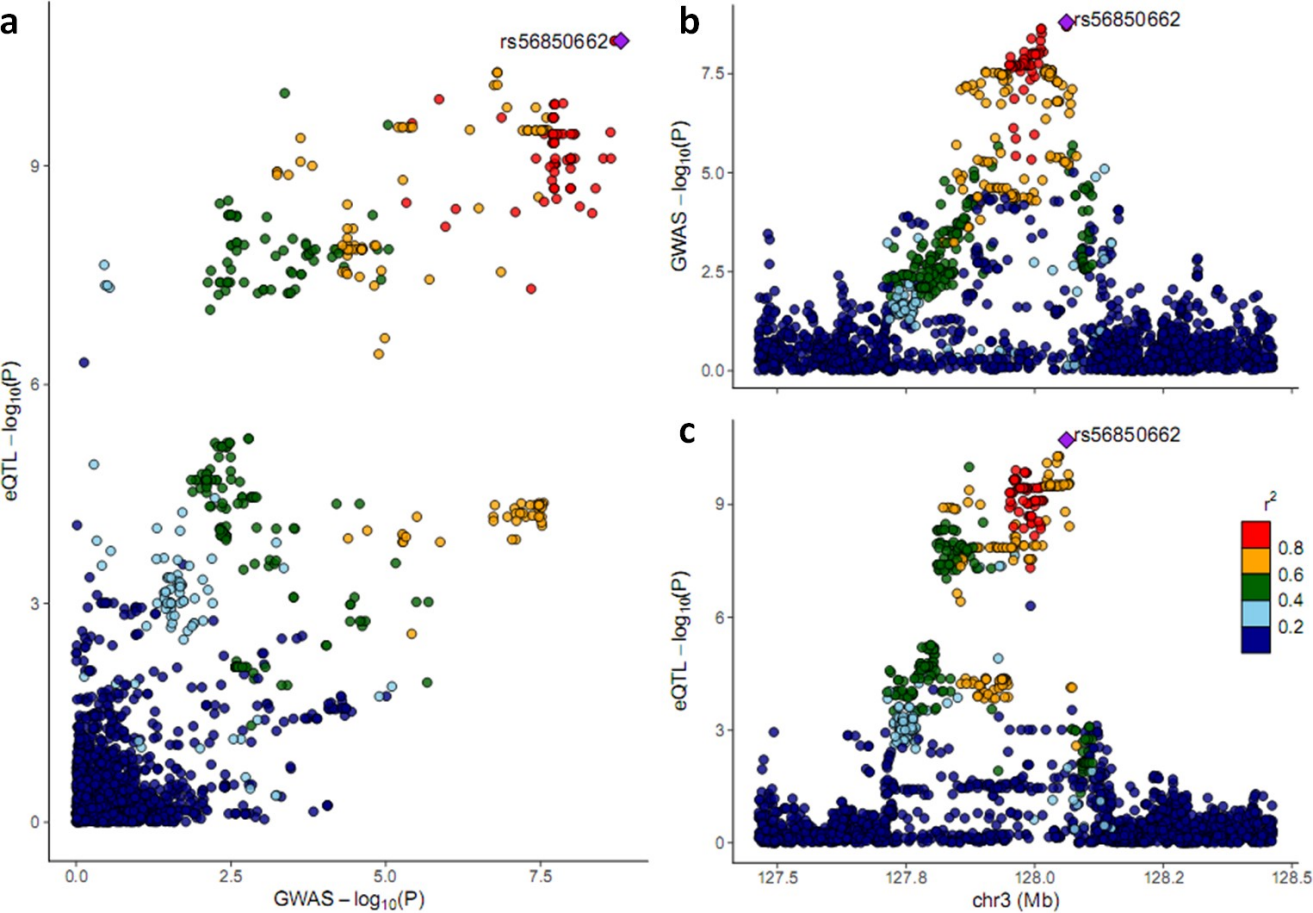
Colocalization plots between Tanzania GWAS and eQTL sites of the *EEFSEC* gene in "Skin—sun exposed" tissue. Note, eQTL results from one representative tissue are shown; similar eQTL signals were observed across multiple tissues and/or cells. The top right plot (b) shows the association results in the Tanzania GWAS; the bottom right plot (c) represents the eQTL results; the left plot (a) shows the colocalization of genetic association and eQTL signals. The SNP indicated by the purple diamond is the SNP for which the African LD information is shown.

GWAS results for each of the 63 facial segments are shown in a composite Manhattan plot (Fig 1B). We identified 189 SNPs across 20 genetic loci showing genome-wide significant (P < 2.5 × 10^−8^) evidence of association with at least one of the 63 facial segments (Tables 1 and S3). Regional association plots for these 20 loci are provided in the supplementary material (S5 Fig). These associations involve facial segments in different quadrants, including seven loci associated with nose-related traits (quadrant 3), four loci associated with eye-related traits (quadrant 2), and three loci associated with segments in more than one quadrant. For nine of the 20 GWAS signals, associations were restricted to localized segments (i.e., at hierarchical level four and five). Of these 20 GWAS signals in Tanzanians, 10 loci (3q28, 4p15.2, 5q14.3, 5q31.1, 7q22.1, 9p21.3, 9q21.33, 10p15.3, 13q13.3, 18q22.1) represent novel associations with facial variation. Moreover, we identified co-localization with eQTLs in fibroblast, skeletal muscle, skin, and adipose tissues/cells for *EEFSEC* (Fig 2).

**Table 1:** Summary of 20 GWAS signals in Tanzania.

											Replication p-values in Euro sample^j^				
Locus	Lead SNP	Chr	Position^a^	A1	A2	MAF	MAF (Euro)^b^	Candidate genes^c^	Best P	Best mod (num mods)^d^	T1^e^	T2^f^	T3^g^	T4^h^	T5^i^
1q22	rs58409393	1	155025307	G	A	0.05	0	ADAM15	1.6E-8	41 (1)	NA^k^	NA	**3.9E-09**	NA	8.8E-05
3p14.3	rs56063440	3	54731374	C	G	0.37	0.28	ESRG	9.7E-9	52 (3)	**1.1E-11**	**6.9E-23**	**2.8E-24**	**1.3E-04**	1.5E-05
3q21.3	chr3:127963189	3	127963189	T	TGC	0.34	NA	EEFSEC	1.5E-11	27 (3)	**1.2E-10**	**1.7E-39**	**9.0E-49**	**2.2E-24**	**4.9E-25**
3q28	rs112643361	3	188438871	G	A	0.10	0	NA	1.8E-8	21 (1)	NA	NA	3.7E-07	NA	7.9E-04
4p15.2	chr4:24163580	4	24163580	G	GAT	0.18	NA	NA	8.9E-9	53 (1)	NA	NA	5.2E-06	NA	2.3E-03
4q31.3	rs9995821	4	154828366	C	T	0.19	0.22	DCHS2	2.5E-22	27 (8)	**2.6E-17**	**5.7E-65**	**5.7E-65**	**1.46E-37**	**1.5E-37**
5q14.3	rs11959408	5	89964298	T	C	0.28	0.34	GRP98	1.1E-8	43 (1)	0.92	0.002	5.5E-06	0.013	2.6E-04
5q31.1	rs113199279	5	134806314	T	G	0.11	0	CXCL14	2.1E-8	28 (1)	NA	NA	7.9E-07	NA	9.5E-06
7q22.1	rs114777090	7	102901689	G	A	0.14	0	NA	8.2E-9	18 (1)	0.49	0.004	3.3E-06	0.26	1.6E-04
9p21.3	rs10122939	9	20300843	G	A	0.28	0.004	MLLT3, FOCAD	3.3E-10	48 (5)	0.02	0.02	4.8E-05	0.21	2.5E-04
9q21.33	rs188502472	9	86936444	T	C	0.03	0.001	NA	2E-9	3 (1)	NA	NA	5.0E-06	NA	1.0E-03
10p15.3	chr10:1582881	10	1582881	AC	A	0.06	NA	NA	2.7E-9	4 (1)	NA	NA	6.6E-07	NA	9.6E-05
10q26.11	rs242980	10	119281243	A	G	0.34	0.17	EMX2	1.5E-11	1 (2)	**2.1E-12**	**4.5E-15**	**1.8E-20**	**4.5E-15**	**1.9E-20**
12q14.3	rs10878346	12	66320873	A	G	0.49	0.25	HMGA2	5.5E-12	1 (4)	**7.9E-10**	**3.0E-15**	**4.0E-19**	**5.4E-13**	**7.6E-18**
12q21.31	rs74112009	12	85808404	A	T	0.46	0.06	ALX1	1.8E-15	30 (6)	**5.9E-05**	**4.7E-14**	**6.6E-29**	**4.7E-14**	**9.9E-23**
12q24.21	rs80243479	12	115356683	C	T	0.04	0	TBX3	2.1E-8	14 (1)	NA	NA	**6.7E-25**	NA	9.4E-04
13q13.3	rs9603276	13	38481292	G	A	0.05	0.4	LINC00571	1.5E-9	11 (1)	0.99	0.0002	3.1E-05	0.1	8.8E-04
13q32.3	rs148390647	13	100542948	G	C	0.01	0	ZIC5	1.4E-8	59 (1)	NA	NA	**2.0E-08**	NA	5.4E-05
18q22.1	rs77926594	18	63466440	A	G	0.02	0	NA	1.6E-8	40 (1)	NA	NA	4.4E-06	NA	9.8E-04
20p11.22	rs16983329	20	22035197	A	G	0.28	0.03	FOXA2	1.5E-8	54 (2)	**2.4E-06**	**1.4E-09**	**4.5E-20**	**1.3E-07**	**8.9E-15**

^a^The chromosome coordinates are based on human genome build 19

^b^MAF of European population were based on 1000 Genome Phase 3 data downloaded from dbSNP; "NA" if there is no frequency data in dbSNP

^c^We determine plausible genes based on functional evidence from previous publication and colocalization result with eQTL

^d^Best mod: The facial segment where the lowest p-value was found (num mods: the number of facial segments where a genome-wide significance was identified)

^e^Association result from the SNP-level testing of the projection of the Tanzania dataset onto the European derived phenotype (T1 in method section)

^f^Association result from the SNP-level look-up of the “best segment” (T2)

^g^Association result from the locus-level look-up of the “best segment” (T3)

^h^Association result from the SNP-level look-up for a qualitatively similar facial segment (T4)

^i^Association result from the locus-level look-up for a qualitatively similar facial segment (T5)

^j^Please find the full result of T1-T5 in S3 Table

^k^The lead SNPs are not available in European samples

**P value highlighted with bold format indicates replication association after Bonferroni correction**

Among these 20 GWAS signals we observed 76 SNPs across six loci that passed the strict threshold of study-wide significance (p < 6.25 × 10^−10^) (see callouts in Fig 1B). Five of these loci (at 3q21.3, 4q31.3, 10q26.11, 12q14.3, and 12q21.31) had previously been identified in the recent facial meta-GWAS of Europeans using the same open-ended phenotyping approach [13], and one locus (9p21.3) was previously identified in a meta-GWAS of the same Europeans using a different phenotyping approach that leveraged facial resemblances among external siblings pairs [35].

### Cell-type-specific enhancer enrichment

**Fig 3:**
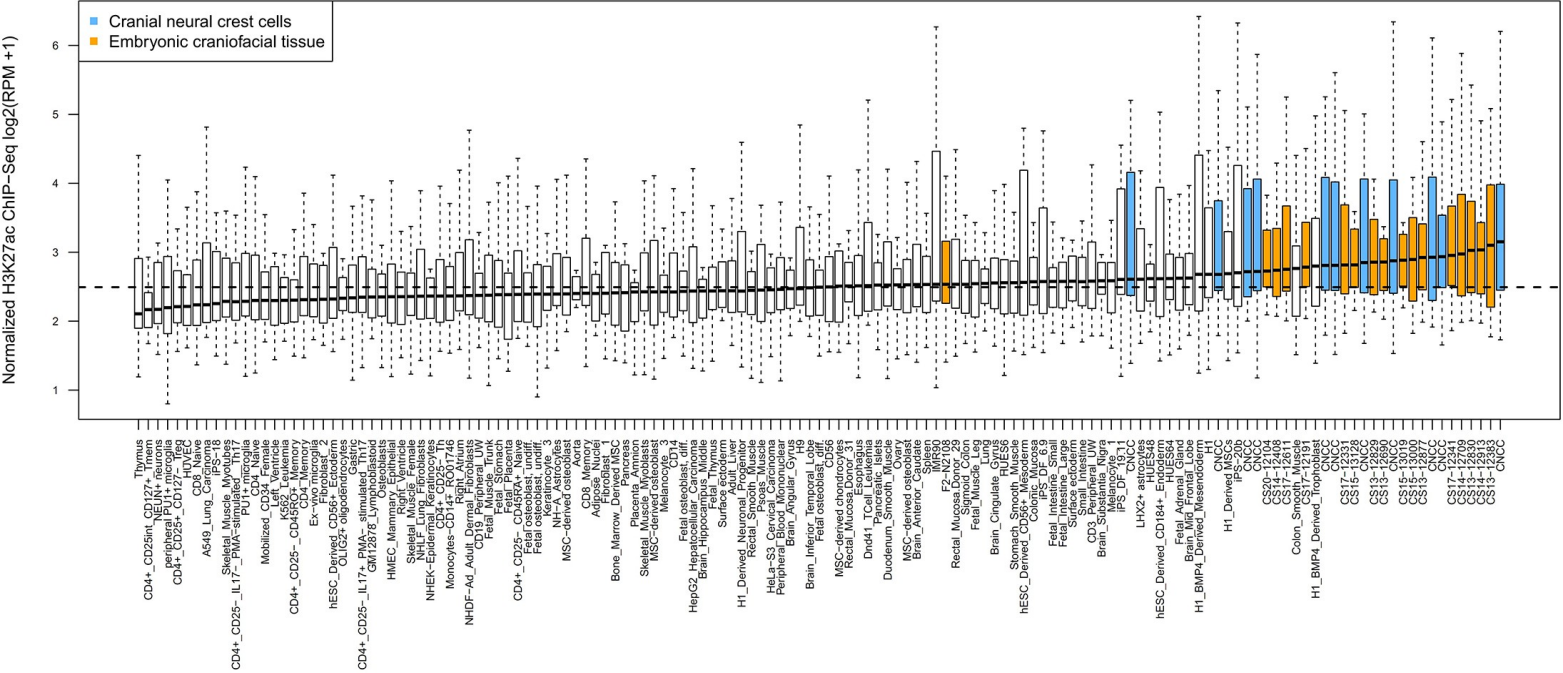
The 20 GWAS loci are enriched for enhancers preferentially active in cranial neural crest cells and embryonic craniofacial tissue. Boxplots indicate H3K27ac signal (log-transformed coverage) in the vicinity of the 20 GWAS loci (within 20kb) in individual samples; cranial neural crest cells and embryonic craniofacial samples are colored blue and orange, respectively. The dashed line at ~2.5 is the median signal across all cell types and tissues.

We explored the cis-regulatory activities of the 20 GWAS loci across more than 100 cell types/tissues. As shown in Fig 3, cranial neural crest cells (CNCCs) and embryonic craniofacial tissues showed the highest H3K27ac signal in the vicinity (within 20kb) of the lead SNPs from the 20 GWAS loci, compared with other cell type/tissues (p = 2.4 × 10^−15^). No predominant enrichment of H3K27ac signal was observed for other primary cell type/tissues. These observations are consistent with previous studies in Europeans [10,13].

We utilized the Roadmap Epigenomics Project data of human embryonic craniofacial samples to identify specific genetic elements that may play a regulatory role during embryonic development nearby the 20 GWAS signals [36]. Among these 20 signals, 11 were located in the vicinity of active enhancers marked by H3K27ac, H3K4me1 or H3K4me2 epigenomic modifications, suggesting their involvement in enhancer activity in craniofacial development. We observed craniofacial-specific enhancer activity at loci 5q31.1 and 12q21.31. Specifically, the signal at 12q21.31 was located near a region with elevated H3K27ac signal at Carnegie stages 17 (CS17) and beyond, and the signal at 5q31.1 was located near a region with H3K27ac, H3K4me1, and H3K4me2 signals from CS13-CS20. Notably, both putative enhancers at 5q31.1 and 12q21.31 contained highly conserved sequences based on base-wise conservation across 100 vertebrates by PhyloP (S6 and S7 Figs).

### Comparisons of East African and European populations

Among the 20 GWAS loci, nine lead SNPs and two high-LD (r^2^ > 0.8) proxy SNPs were available in the European dataset. The remaining nine SNPs were not available in the European cohort due to the low minor allele frequency in the European population. Seven of the 11 lead SNPs (i.e. rs56063440, chr3:127963189, rs9995821, rs242980, rs10878346, rs74112009 and rs16983329) replicated under the projected phenotype approach (T1), indicating consistent genetic effects in the projected African-trait facial segment (Table 1). SNP-level look-ups for qualitatively similar facial segments (T4) and for the “best segments” (T2), showed that the same seven SNPs were associated with facial variation in the previous GWAS of Europeans (Table 1). Of the four lead SNPs that failed to replicate, two (rs114777090 and rs10122939) had very low frequencies in the European cohort (MAF<0.01), which may account for the lack of signal, and two (rs11959408 and rs9603276) had high frequencies in both populations, suggesting that differences in allele frequency, alone, are unable to explain the lack of signal. The locus-level replication approaches (T3 and T5) indicated that an additional three loci, 1q22, 12q24.21 and 13q32.3, were associated with facial variation in European samples, albeit with different associated SNPs. The S8 Fig presents the SNP-level and locus-level replication in Europeans.

**Fig 4:**
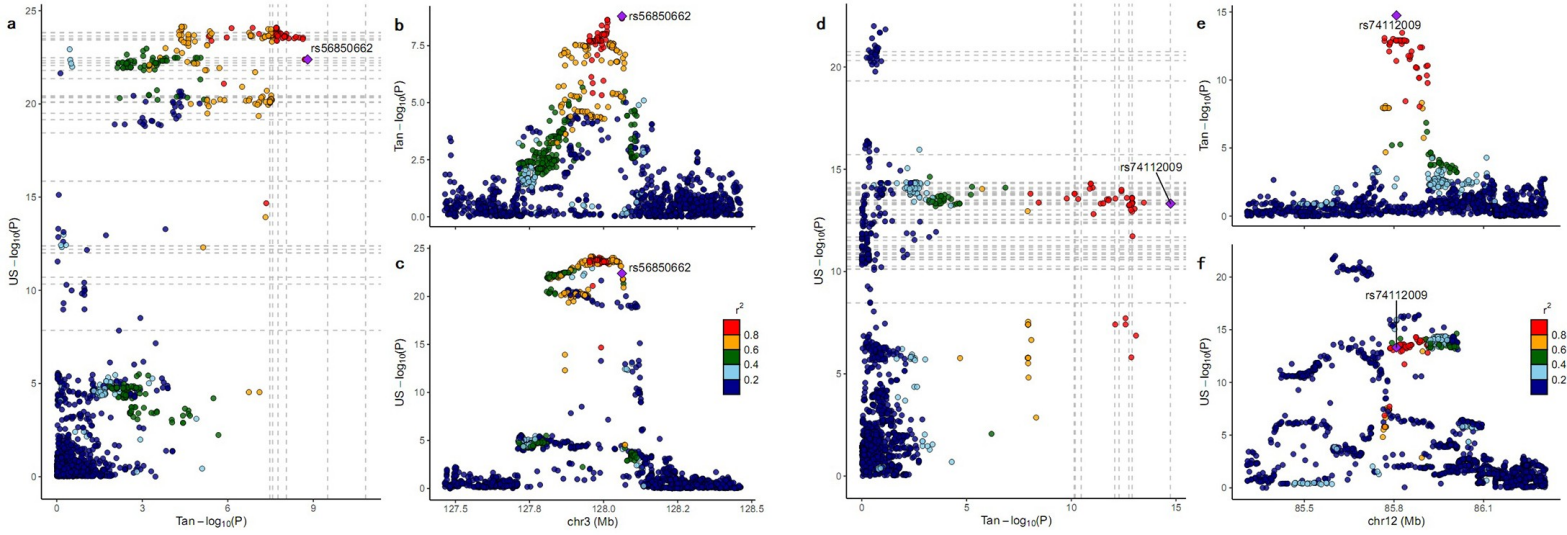
LocusCompare visualizations of colocalization between Tanzania GWAS and European GWAS at (a-c) 3q21.3 and (d-f) 12q21.31. The top right plots (b and e) show the association results in the Tanzania GWAS; the bottom right plots (c and f) represent the corresponding results in the European GWAS; the left plots (a and d) are visualizations of colocalization. For each locus, the SNP indicated by the purple diamond is the SNP for which the LD information is shown, with African LD structure indicated in the colocalization plot. The vertical gray dashed lines indicate the p-values of SNPs from the Tanzania GWAS that were unavailable in the European GWAS; the horizontal gray dashed lines indicate the p-values of SNPs from the European GWAS that were unavailable in in the Tanzanian GWAS.

We performed co-localization analysis for the 20 GWAS loci using the summary statistics of qualitatively similar facial segments from European GWAS. Notably, our findings suggested that signals at 3p14.3, 4q31.3, 10q26.11, and 12q14.3 may share the same causal variants in African and European populations (S5 Fig). For the rest of the loci, the co-localization plots showed more complicated scenarios (Fig 4). For example, at 3q21.3, the peak SNP in the European GWAS was located within a broad LD block of approximately 300Kb, with many associated SNPs highly correlated with each other, which poses a challenge in determining the causal variant underlying the association. Combining the signal in the Europeans with the signal in Tanzanians provided a more finely mapped result, suggesting a specific casual variant, rs56850662. This is the same variant that co-localized with the eQTL signal for *EEFSEC*, suggesting a possible role for *EEFSEC* in craniofacial morphology (Figs 2 and S5). For locus 12q21.31, only a subset of SNPs showing association in Europeans co-localized with the Tanzania signal, identifying a shared associated variant, and suggesting that more than one genetic element is related to mouth and upper lip morphology in Europeans.

In addition to exploring the effects of the Tanzania GWAS signals in Europeans, we also examined 203 association signals across 138 genetic loci previously identified in the GWAS of Europeans for their associations with the corresponding projected facial trait in the Tanzania sample. Of the 203 lead SNPs, 195 were available in the Tanzania sample. Among tested SNPs, 12 were associated with the same facial phenotype (T1) in the Tanzania sample, indicating consistent effects across different ancestry groups (S4 Table). The SNP-level look-ups for the “best segment” revealed eight significant associations, including four signals that were not identified by the projected phenotype approach (T2).

Beyond testing specific lead SNPs for replication, we also explored evidence of association in the Tanzania cohort for all SNPs across the 203 signals in case different variants in these regions were associated in the different ancestry groups. For the candidate locus scan, 13 additional signals were associated under the significance threshold of 0.05 divided by 203 and then divided by the effective number of independent SNPs at each locus. However, these loci were associated with different facial traits (i.e., modules representing different regions of the face) across the different populations, suggesting the presence of more than one regulatory element within a genetic locus potentially affecting different facial segments. The shared association signals in African and European populations are displayed in the Miami plot (S8 Fig).

### Test of previously reported facial-associated loci

As shown in S1 Table, genetic association at 112 loci have been reported in the previous 15 studies of *a priori* landmark-based and qualitative (e.g., cleft chin, cheek dimple) phenotypes, of which five and 11 loci were replicated based on (T2) and (T3), respectively (S1 Table). Furthermore, 12 out of these 112 loci had previously shown associations in at least two independent GWAS, with genetic effects of *PAX3*, *PAX1*, *SOX9*, *TBX3* and the HOXD cluster on different facial traits reported in European, Asian, and Latin American populations. Among these 12 genetic loci that were identified by multiple GWASs, *CACNA2D3*, *DCHS2*, *TBX3*, *PAX1*, and the HOXD cluster were significantly associated with facial variation in the Tanzania cohort after Bonferroni correction (S1 Table). In contrast, *SOX9* and *PAX3* have been reported across populations (S1 Table), whereas neither of these genes showed association in Tanzania cohort.

## Discussion

In this study, we performed a GWAS of multidimensional facial traits in a sample of 2,595 unrelated healthy children and adolescents of African ancestry. We identified 20 genetic loci that were associated with normal facial variation at a genome-wide significance threshold of p < 2.5 × 10^−8^. Of these, six loci (3q21.3, 4q31.3, 9p21.3, 10q26.11, 12q14.3 and 12q21.31) surpassed our more conservative threshold for study-wide significance (p < 6.25 × 10^−10^). The locus 9p21.3 was not identified in the previous meta-GWAS of data-driven facial traits in Europeans, but a nearby SNP (~20Kb to the peak SNP in Tanzania) was identified in a GWAS of different traits derived from resemblance between siblings and projected into the same cohort of Europeans. The lead 9p21.3 SNP, rs10122939, is in the vicinity of the *MLLT3* gene, a crucial regulator of human haematopoietic stem cells associated with acute leukemia. It remains unclear how the genetic findings of *MLLT3* may relate to facial morphology. Another candidate at 9p21.3 is *FOCAD*, located ~350Kb downstream of the peak SNP, which is a potential tumor suppressor highly expressed in brain tissues. Haaland et al. identified a parent-of-origin interaction effect between *FOCAD* and maternal smoking contributing to cleft lip [37]. In order to determine the possible roles of genes at this locus on facial traits, subsequent functional studies are needed.

For the locus at 3q21.3, though different lead SNPs were observed in the Tanzania and European GWASs, co-localization analysis narrowed this down to a single intronic variant, rs56850662, in the *EEFSEC* gene, suggesting that this SNP drives the association with nose and lip morphology in both populations (S5 Fig). Furthermore, this variant co-localized with an eQTL signal for *EEFSEC*, supporting the role of *EEFSEC* as a candidate gene for facial morphology.

4q31.3 is a facial-associated locus with accumulated genetic evidence that indicates its role in face formation [3,10,17]. The present study replicated the association with normal nasal variation in an African population, demonstrating its involvement in the genetic architecture of nose morphology across populations. The nearest gene to the peak SNP in the Tanzania GWAS is *SFRP2*, encoding the Secreted Frizzled Related Protein 2. *SFRP2* functions as modulator of Wnt signaling, whose overexpression can induce the limb outgrowth defect [38,39]. Craniofacial defects, limb outgrowth defects, and extra digits were reported in *Sfrp2-/-* mutant mice [40,41]. Another interesting candidate at 4q31.3 is the *DCHS2* gene, located ~300Kb downstream of the peak SNP. *DCHS2* encodes a calcium-dependent cell-adhesion protein, known as a key partner in the Fat-Dachsous signaling pathway that coordinates cartilage differentiation and polarity during craniofacial development [42].

The other three loci showing study-wide evidence of association in Tanzanians, 10q26.11, 12q14.3, and 12q21.31, were reported by previous GWASs in European or Asian samples. Our findings revealed that these associations were shared across different ancestry groups. Together with functional evidence [7,43–45], plausible candidate genes within these loci (i.e. *HMGA2* at 12q14.3, *EMX2* at 10q26.11, and *ALX1* at 12q21.31) may play critical roles in craniofacial development. Further investigation is needed to establish their functionality and the mechanisms through which they impact normal facial variation.

In addition to the study-wide significant loci, we also identified 10 novel loci in Africans at genome-wide significance, some of which were near genes having known involvement in craniofacial development. For example, *CXCL14*, which was associated with variation in the eye region, plays a critical role in ocular tissues during development [46–48]. Knockdown of *CXCL14* led to eyelid and mandibular defects in about a third of chick embryos, which is consistent with its expression in eyelid ectoderm and the first branchial arch [47]. We also identified new signals near biologically plausible genes at previously reported loci. For example, the association signal at 13q32.3, near the zinc finger genes, *ZIC2* and *ZIC5*, was also associated with variation in the eye region. *ZIC5* is expressed in the developing eyes in *Xenopus* embryos, and loss of *zic5* in mice causes craniofacial anomalies [49,50]. Moreover, mouse and *Xenopus* models have shown that Zic5 protein is involved in the generation of neural crest tissue [49,51]. Because this association in Tanzanians was moderately far (about 430kb) from the previously identified signal in Europeans and affected different regions of face (eyes in Tanzanians vs. forehead in Europeans) [13], it is unclear whether it should be considered a novel, separate locus or a new association signal at the same locus. In any case, these associations with biologically plausible candidates observed in Africans require additional research to determine whether they are ancestry-specific.

Of the 20 GWAS signals identified in the Tanzania cohort, seven showed associations in Europeans, suggesting trans-ancestry genetic effects on normal facial variation. Notably, these loci showed significant associations with the same facial segments among populations, which improved the reliability of the findings. For some of these loci, the GWAS in Europeans identified more global (broader) effects on the face compared to Tanzanians. For example, the peak SNP at locus 3q21.3 was associated with shape of nose and upper lip in Europeans, but only associated with the shape of nose in Tanzanians. The other 13 loci did not replicate in Europeans. For 11 of these, the top SNP had low allele frequency in the European cohort (MAF<0.01), suggesting these differences may be partly attributable to the allele frequency differences.

Our GWAS in Tanzanians not only uncovered novel loci and candidate genes related to facial morphology, but also advanced our understanding of previously identified loci in Europeans. Several loci (such as 3q21.3 and 12q21.31) showed associations with facial morphology across different populations. However, due to the strong LD in Europeans, association was detected across a broad genomic region (~300Kb), posing a challenge for identifying the likely causal variant at these loci. In conjunction with the European results, co-localization utilizing the GWAS in Tanzanians provided a more fine-mapped association. Given human evolutionary history, African populations are characterized by a greater level of genetic diversity and less LD among loci compared with European populations. Because of the specific LD structure, the GWAS in Africans offered valuable insights relevant to the genetic factors that contribute to normal facial variation. That said, only a fraction of the loci originally identified in Europeans showed evidence of association in the Tanzanian cohort, which we postulate may be partly attributable to the population differentiation. Of the 203 European signals, more than half of the peak SNPs had substantial allele frequency differences between European and African populations (MAF difference >0.1), which would impact the power to detect associations. Furthermore, the low rate of replication may also be due to insufficient power in the Tanzanian sample due to the smaller sample size and the stricter p-value threshold for declaring significance. For these reasons, we caution that lack of replication across populations should not be taken as conclusive evidence that a signal is population-specific.

As a whole, our results provided a glimpse into the developmental origins of facial variation. Given that CNCCs are a group of embryonic cells that give rise to most facial structures and arise at 3–6 weeks of human gestation, if the associations with facial shape captured by our GWAS are due to effects occurring early in embryogenesis then we expect activity of facial-associated loci to be observed in CNCCs. We not only showed the enrichment of facial-associated variants in CNCCs and embryonic craniofacial tissues, but also identified two facial-associated loci overlapping with putative craniofacial-specific enhancer activity. The regulatory elements lie in sequences highly conserved across vertebrates, indicating their possible functional importance. Specifically, the GWAS signal at 12q21.31 associated with facial variation in the eye region overlaps with a craniofacial-specific enhancer that is active beginning at Carnegie Stage 17, when the eyelids begin to form [52].

Continuing challenges in researching the genetics of facial shape include the heterogeneity across diverse human populations, inconsistent phenotypic strategies, and the influence of environmental factors. We performed the same data-driven facial-segmentation phenotyping strategy in both Tanzanian and European cohorts, which leads to differences between studies in the exact facial phenotypes. Though overall quite similar, one major difference in the Tanzania facial segmentation was the emergence of an eye-related sub-quadrant, which was absent from the facial segmentation in Europeans. As a result of the presence of eye-related segments, we identified novel loci associated with facial shape around the eyes. The eye-related segmentation may be partly attributed to the different approach to collect 3D images. For the European cohorts, 3D facial images were obtained while participants’ eyes were fully opened during image capture, whereas for the Tanzania cohort, participants were not required to open their eyes while collecting the 3D image. To determine the effect of variation in open vs. closed eyes during imaging, we included the predicted open/closed state of the eyes as an additional covariate in our facial model and re-ran the segmentation and genome scans. Segmentation was largely similar with the exception of the eye-related sub-quadrant, which was absent in the eye-adjusted analysis. Likewise, genetic association results were similar, with the exception of signals specific to the eye-related modules. See S1 Appendix describing these methods and results.

Another potential limitation of the study is the restricted age range of our sample, comprising mostly children and adolescents whose faces are still developing. While our covariate adjustments adequately accounted for mean effect of age and age^2^ on facial variation, the sample may include some children whose faces are under- or over-developed for their chronological age. This deviation from the average growth trajectory may decrease the signal to noise ratio, thus reducing power to detect genetic associations, or may represent a timing-specific aspect of facial variation, possibly under genetic control. Despite this limitation, it is important to acknowledge that failure to completely account for the effects of age variation on facial traits is unlikely to result in false positive genome-wide signals.

This study is a re-analysis of the dataset originally reported in Cole et al. [18] and represents the first effort to apply a global-to-local phenotyping method to a GWAS of Africans. Using the phenotypic method, we have greatly expanded the number of discovered loci that were associated with normal-range facial traits in an African population. Of note, none of the identified signals overlapped the two previously identified loci in this cohort using landmark-based size and shape phenotypes [18]. Considering that the two studies used the same GWAS cohort, but different phenotyping strategies that capture altogether different aspects of facial variation, the difference in results is not unexpected. In particular, Cole et al. [18] reported associations of SNPs in *SCHIP1* and *PDE8A* with facial size, whereas in the present study we did not investigate size or allometry phenotypes; instead we adjusted our phenotypes for facial size. Moreover, the global-to-local phenotyping approach employed in this study was developed specifically because landmark-based phenotypes, such as those used in Cole et al. [18] do not as fully capture shape variation as do the global-to-local modules. That the observed associations differ by phenotyping approach is consistent with what has previously been reported in European samples. Previous GWAS of global-to-local facial shape modules yielded more but altogether different signals than traditional landmark-based phenotypes in the same sample [8,10]. Taken together, these observations in both European and African datasets suggest that fundamentally different aspects of the underlying genetic architecture of facial variation are being captured by two phenotypic strategies.

While verifying the association of some previously reported loci, we provide new evidence that these loci contribute to facial shape variation across populations. Among these replicating loci, several genes, such as *EEFSEC*, *SFRP2*, *EMX2*, *ALX1* and *HMGA2*, are biologically plausible candidates that play important roles in embryological facial tissues. In addition, we revealed 10 new genetic loci that passed the threshold for genome-wide significance, and which are candidates for future replication studies. These findings improve our understanding of genetic and biological basis underpinning the diversity of human facial structure and may offer valuable insights into to biological mechanisms responsible for craniofacial morphogenesis and dysmorphology. Additional genetic replication and experimental validation will be required to verify the handful of newly identified genes/loci with unclear roles in craniofacial development.

## Supporting information

S1 Fig Age distribution among participants that were retained for the genetic analysis. (TIF)

S2 Fig Principal component analysis (PCA) scatterplot illustrating the population structure of 2,595 unrelated participants that were retained for the genetic analysis. (TIF)

S3 Fig Color-coded facial segmentations in Tanzanians (left) and Europeans (right). The entire face (red) is partitioned in to outer face (orange) and midface (cyan), and further partitioned in more localized regions representing the lower face (magenta), upper face (salmon), nose (blue), and mouth and eyes (green).
(TIF)

S4 Fig Workflow of the study. (TIF)

S5 Fig LocusZoom (LZ) plots, colocalization plot and a polar dendrogram showing global-to-local effect for each of 20 GWAS signals. Three LZ plots represent the genetic associations in (a) the best facial segment in Tanzania, (b) a comparable facial segment in Europeans, and (c) the best facial segment in Europeans. (d) A colocalization plot between the Tanzania GWAS and European GWAS at the chromosome 1q22 locus; (e) the association results in the Tanzania GWAS; (f) the association results in the European GWAS. (d-f) The SNP indicated by the purple diamond is the SNP for which the LD information is shown, with African LD structure indicated in the colocalization plot. The vertical gray dashed lines indicate the p-values of SNPs from the Tanzania GWAS that were unavailable in the European GWAS; the horizontal gray dashed lines indicate the p-values of SNPs from the European GWAS that were unavailable in in the Tanzanian GWAS (g) A polar dendrogram showing the global-to-local effect in Tanzania GWAS. Facial segments with a p-value lower than the genome-wide threshold (p = 2.5 × 10−8) are circled in black.
(PDF)

S6 Fig (a) regional association plot for the signal at 5q31.1. (b) UCSC genome browser custom tracks for the 5q31.1 region, in which the yellow colored bars represent enhancer activity and the green colored bars represent Tx (Strong_transcription).
(TIF)

S7 Fig (a) regional association plot for the signal at 12q21.31. (b) UCSC genome browser custom tracks for the 12q21.31 region, in which the yellow/orange colored bars represent enhancer activity; the purple colored bars represent PromBiv (Bivalent Promoter); the grey colored bars represent ReprPC (Repressed_PolyComb).
(TIF)

S8 Fig Miami plot showing (upper) Tanzanian and (lower) European GWAS results. The dashed blue line in each panel indicates the genome-wide significance threshold, and the red solid line indicates the study-wide significance cutoff. In each panel, the cyan and red colored points, respectively, represent signals showing locus-level and SNP-level evidence of replication in the alternate cohort.
(TIF)

S1 Table Replication of previously reported landmark-based and qualitative trait associations in the Tanzania dataset. (XLSX)

S2 Table The number of principal components retained after parallel analysis for each facial segment. (XLSX)

S3 Table Full results 20 GWAS signals in Tanzania. (XLSX)

S4 Table In silico replication of European hits in the Tanzania dataset. (XLSX)

S1 Appendix Prediction of open vs. closed eyes and sensitivity analysis exploring the effect of open vs. closed eyes on the facial segmentation and genome-wide association analyses. (PDF)
