# Supplementary figures and images for: Genome scans of facial features in East Africans and cross-population comparisons reveal novel associations
Source: PLoS Genet. 2021 Aug 19;17(8):e1009695. doi: 10.1371/journal.pgen.1009695 (PMC8375984; doi:10.1371/journal.pgen.1009695)

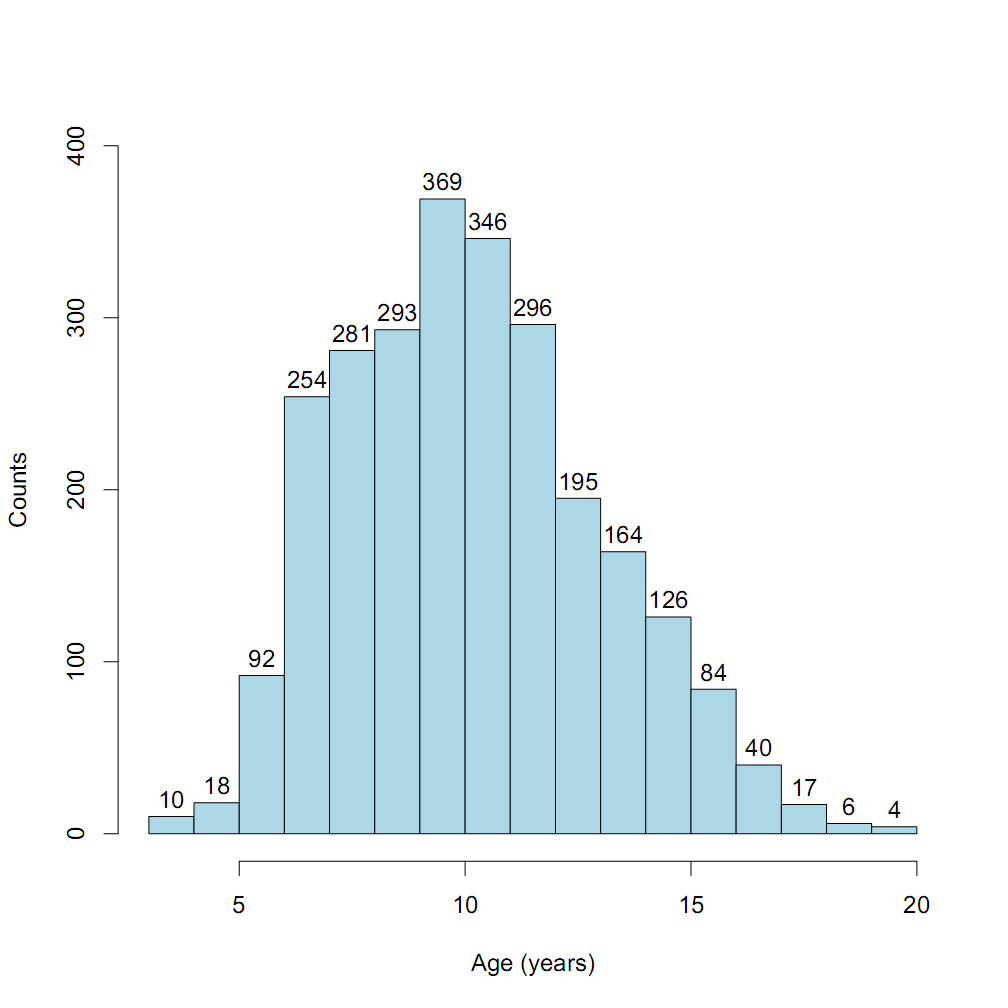

Supplement: S1 Fig — (TIF) [file pgen.1009695.s001.tif]

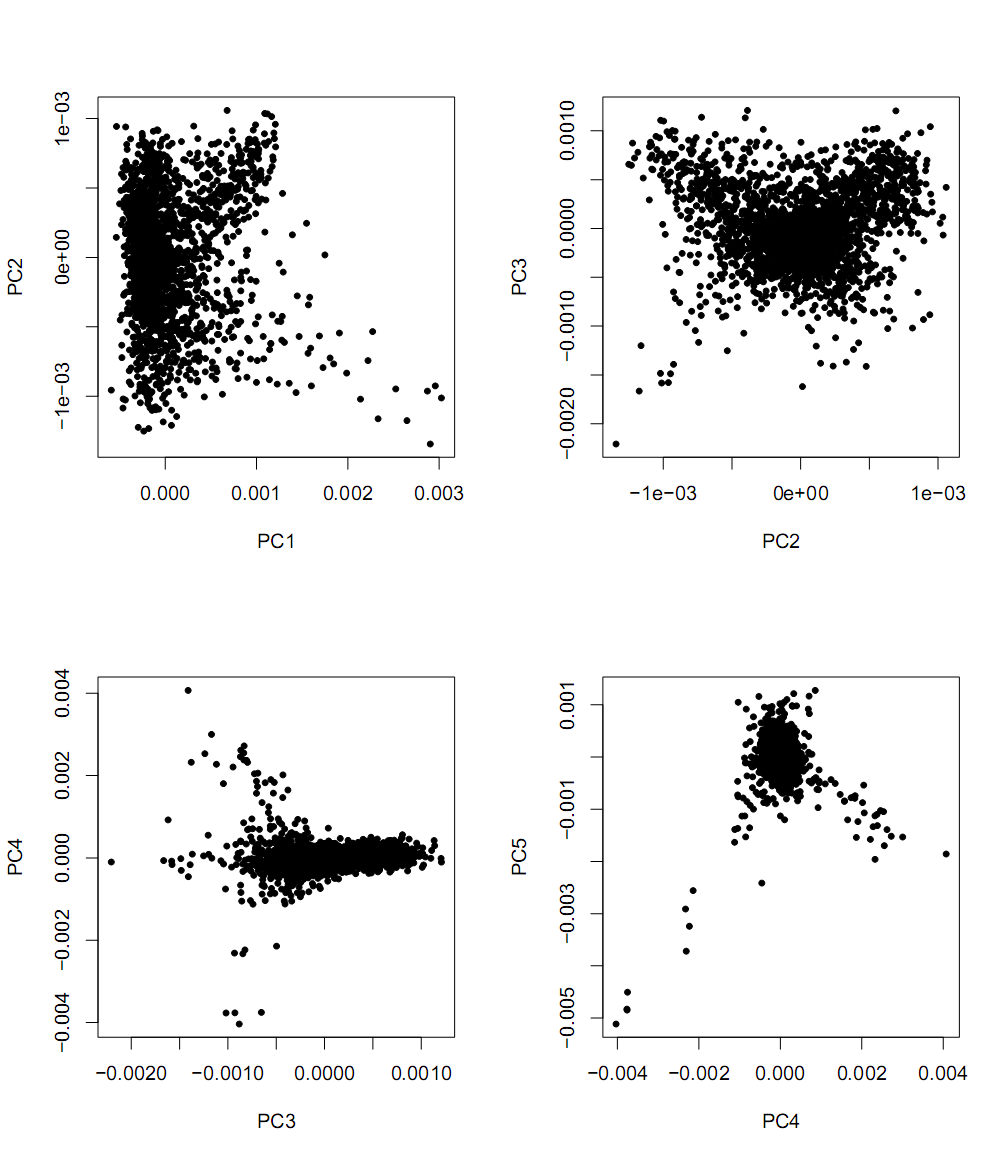

Supplement: S2 Fig — (TIF) [file pgen.1009695.s002.tif]

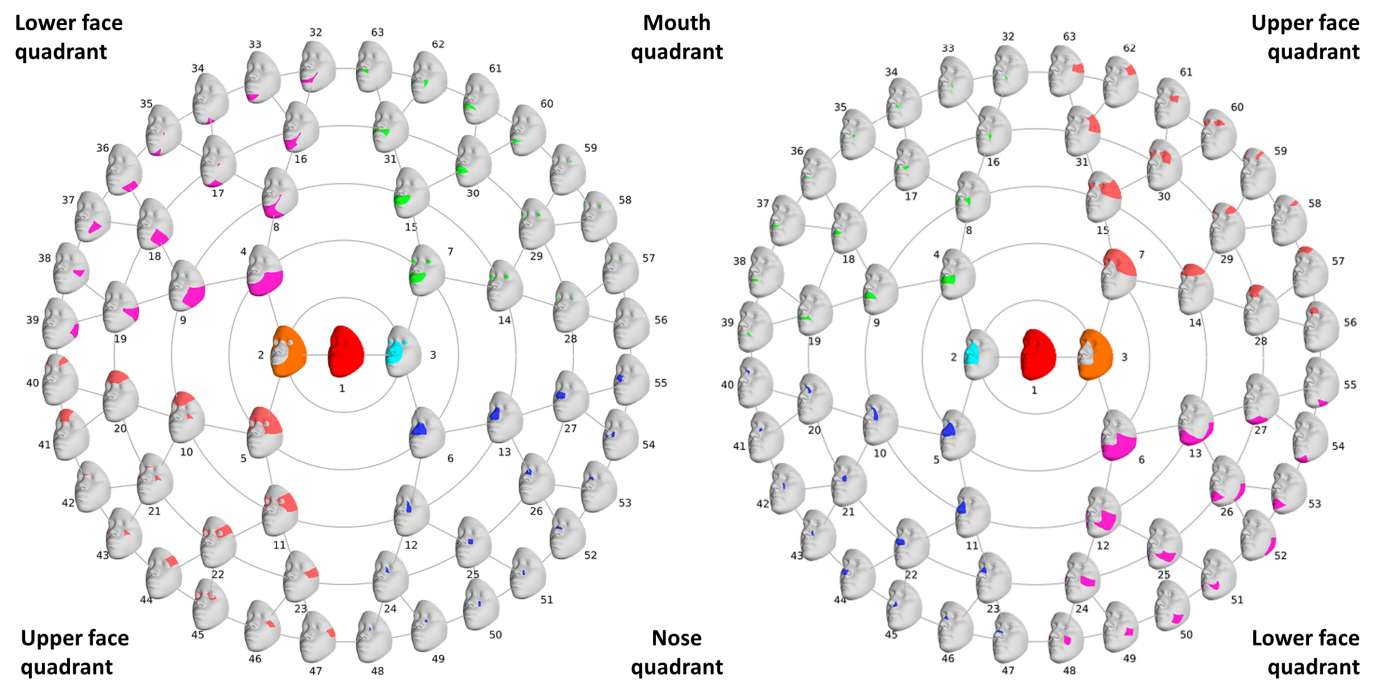

Supplement: S3 Fig — Color-coded facial segmentations in Tanzanians (left) and Europeans (right). The entire face (red) is partitioned in to outer face (orange) and midface (cyan), and further partitioned in more localized regions representing the lower face (magenta), upper face (salmon), nose (blue), and mouth and eyes (green). (TIF) [file pgen.1009695.s003.tif]

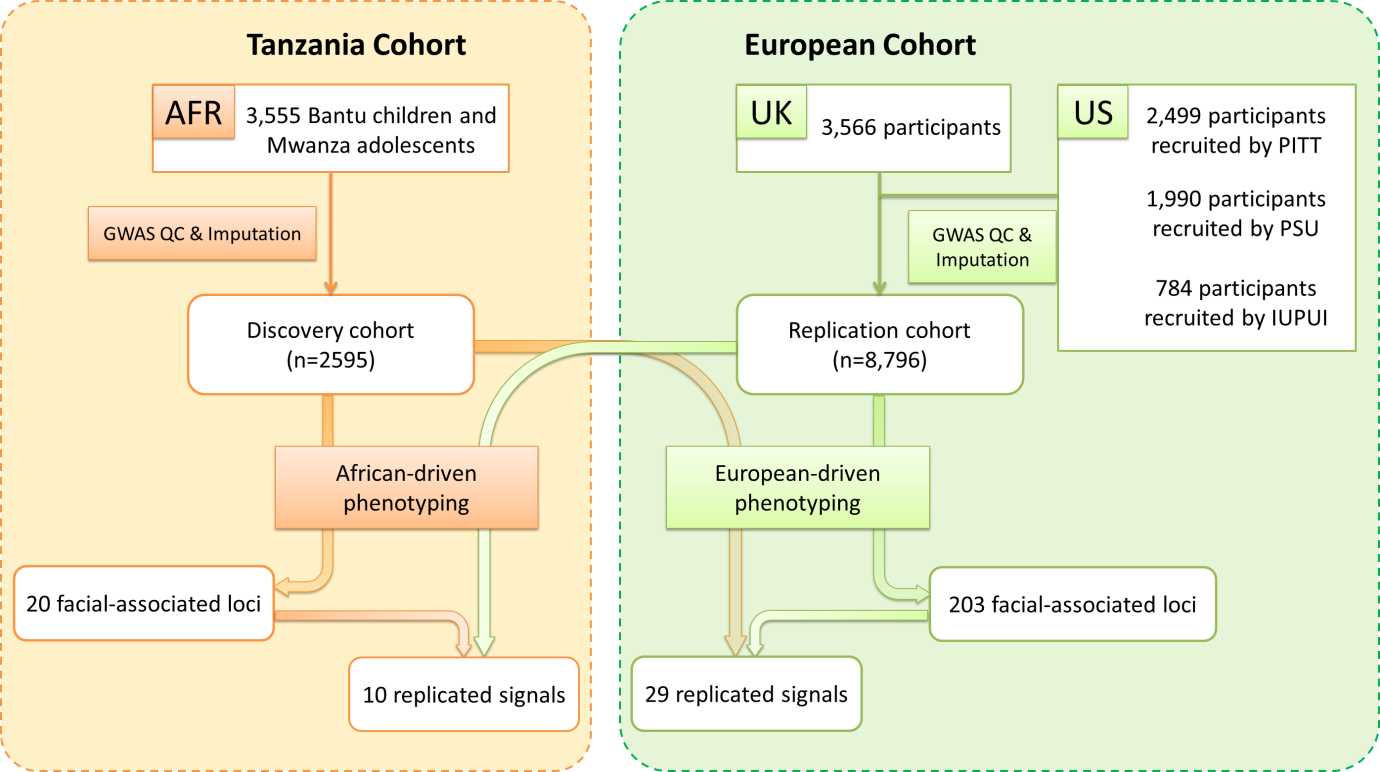

Supplement: S4 Fig — (TIF) [file pgen.1009695.s004.tif]

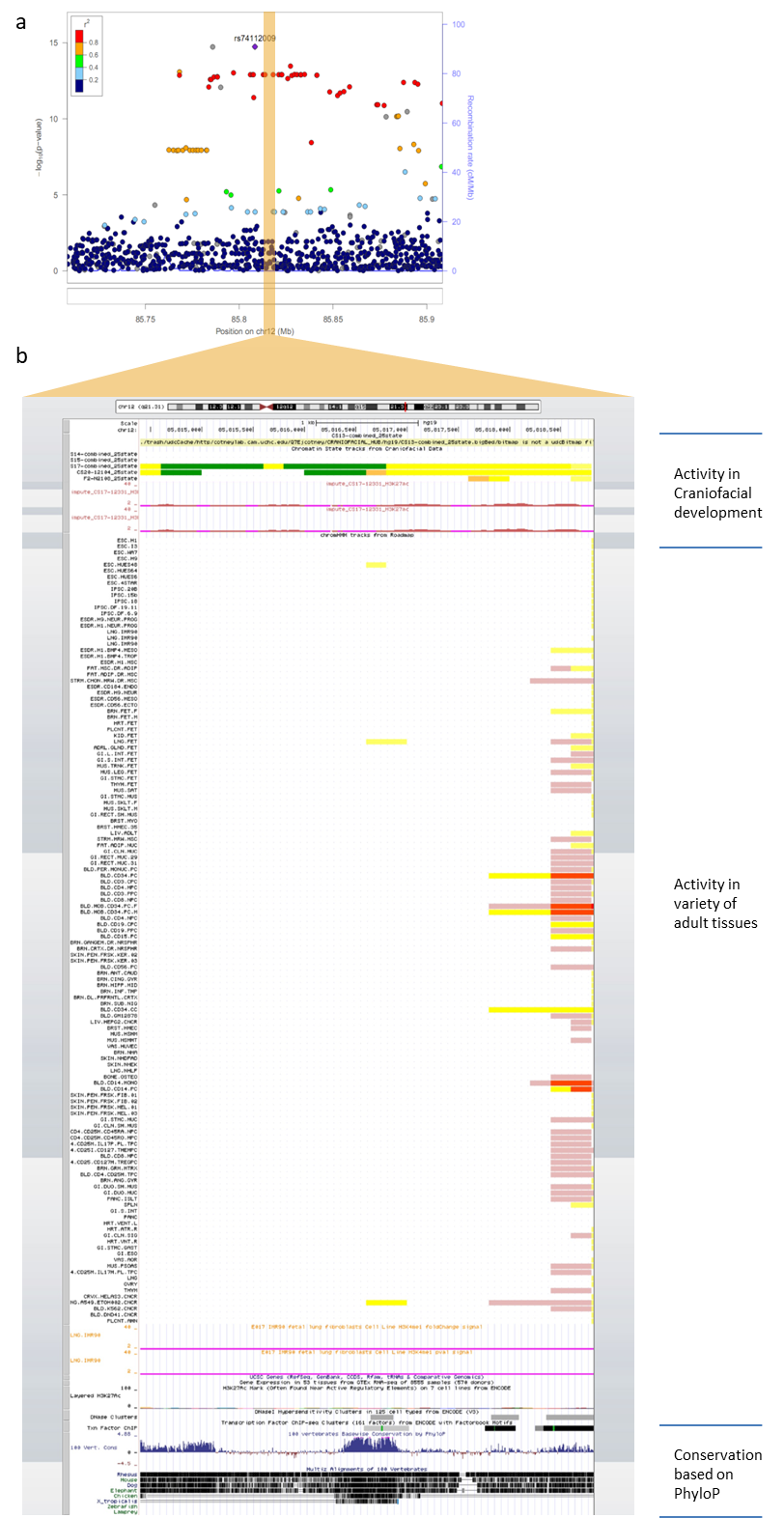

Supplement: S6 Fig — (a) regional association plot for the signal at 5q31.1. (b) UCSC genome browser custom tracks for the 5q31.1 region, in which the yellow colored bars represent enhancer activity and the green colored bars represent Tx (Strong_transcription). (TIF) [file pgen.1009695.s006.tif]

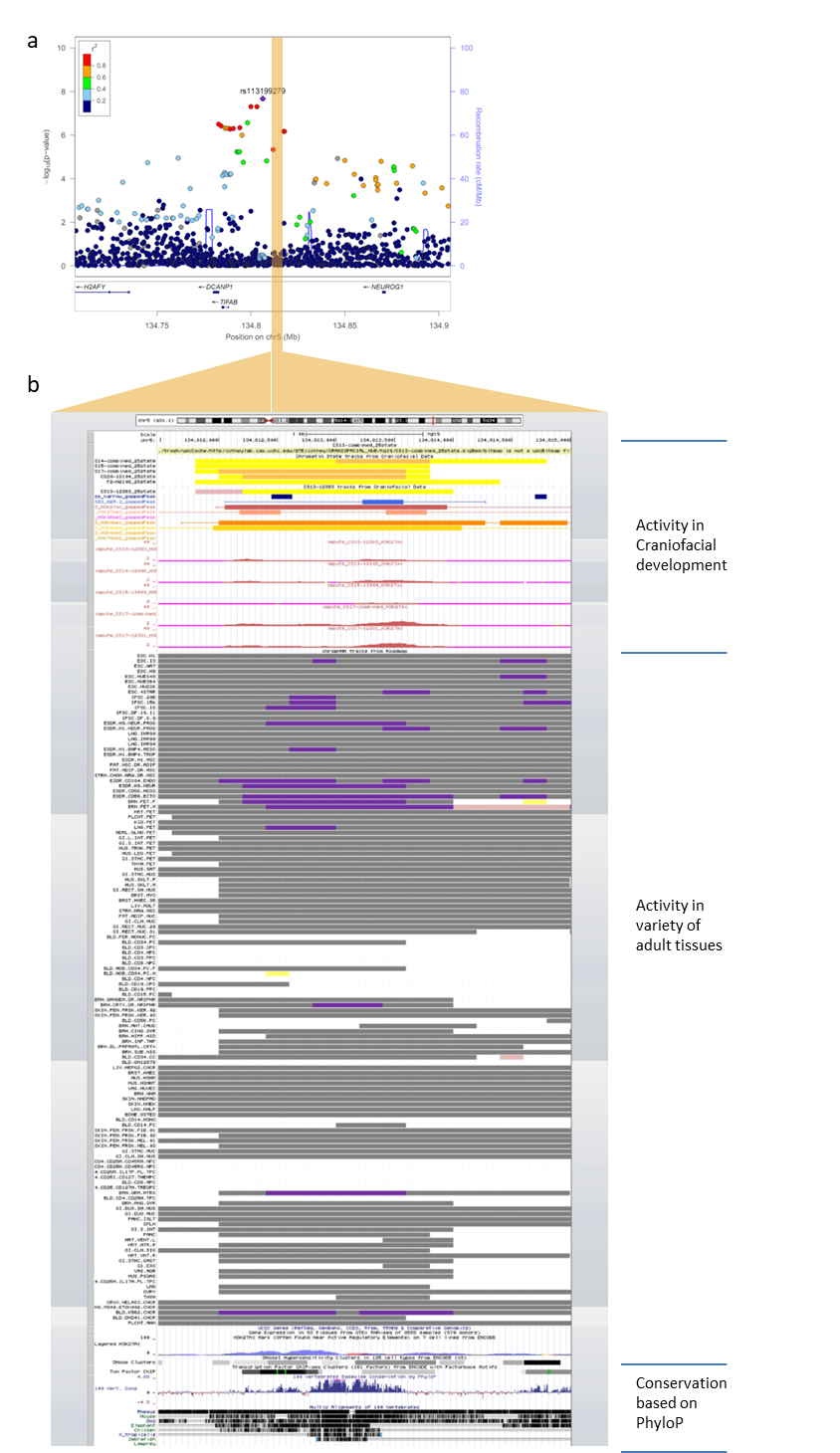

Supplement: S7 Fig — (a) regional association plot for the signal at 12q21.31. (b) UCSC genome browser custom tracks for the 12q21.31 region, in which the yellow/orange colored bars represent enhancer activity; the purple colored bars represent PromBiv (Bivalent Promoter); the grey colored bars represent ReprPC (Repressed_PolyComb). (TIF) [file pgen.1009695.s007.tif]

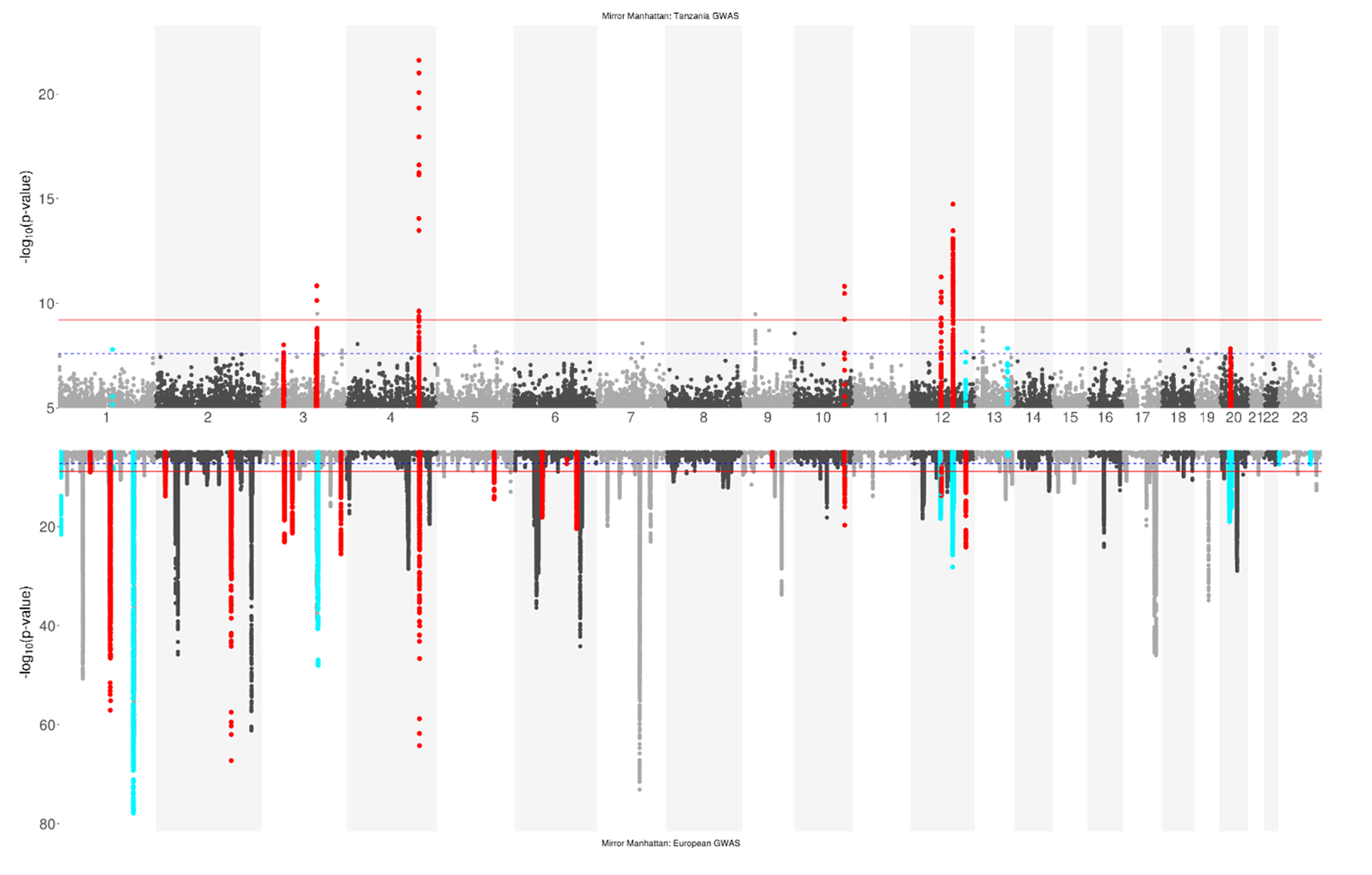

Supplement: S8 Fig — Miami plot showing (upper) Tanzanian and (lower) European GWAS results. The dashed blue line in each panel indicates the genome-wide significance threshold, and the red solid line indicates the study-wide significance cutoff. In each panel, the cyan and red colored points, respectively, represent signals showing locus-level and SNP-level evidence of replication in the alternate cohort. (TIF) [file pgen.1009695.s008.tif]
