# Supplementary material for: Genome scans of facial features in East Africans and cross-population comparisons reveal novel associations
Source: PLoS Genet. 2021 Aug 19;17(8):e1009695. doi: 10.1371/journal.pgen.1009695 (PMC8375984; doi:10.1371/journal.pgen.1009695)

GWAS signal at 1q22

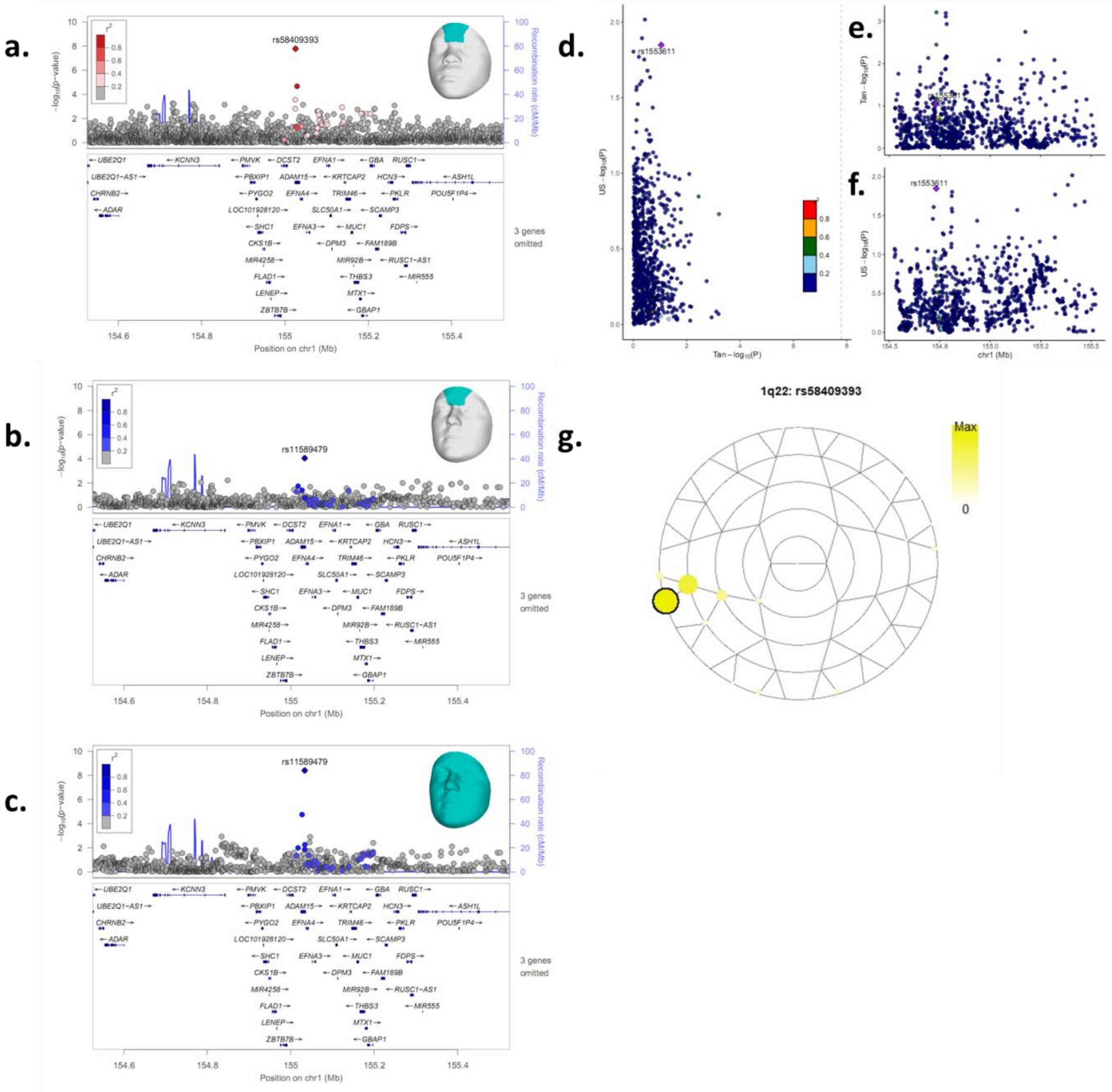

Supplementary Figure S5. LZ plots, colocalization plot and a polar dendrogram showing global-to-local effect for each of 20 GWAS signals. Three LZ plots represent the genetic associations in (a) the best facial segment in Tanzania, (b) a comparable facial segment in Europeans, and (c) the best facial segment in Europeans. (d) A colocalization plot between the Tanzania GWAS and European GWAS at the chromosome 1q22 locus; (e) the association results in the Tanzania GWAS; (f) the association results in the European GWAS. (d-f) The SNP indicated by the purple diamond is the SNP for which the LD information is shown, with African LD structure indicated in the colocalization plot. The vertical gray dashed lines indicate the p-values of SNPs from the Tanzania GWAS that were unavailable in the European GWAS; the horizontal gray dashed lines indicate the p-values of SNPs from the European GWAS that were unavailable in in the Tanzanian GWAS (g) A polar dendrogram showing the global-to-local effect in Tanzania GWAS. Facial segments with a p-value lower than the genome-wide threshold ($p = 2.5 \times 10^{-8}$) are circled in black.

GWAS signal at 3p14.3

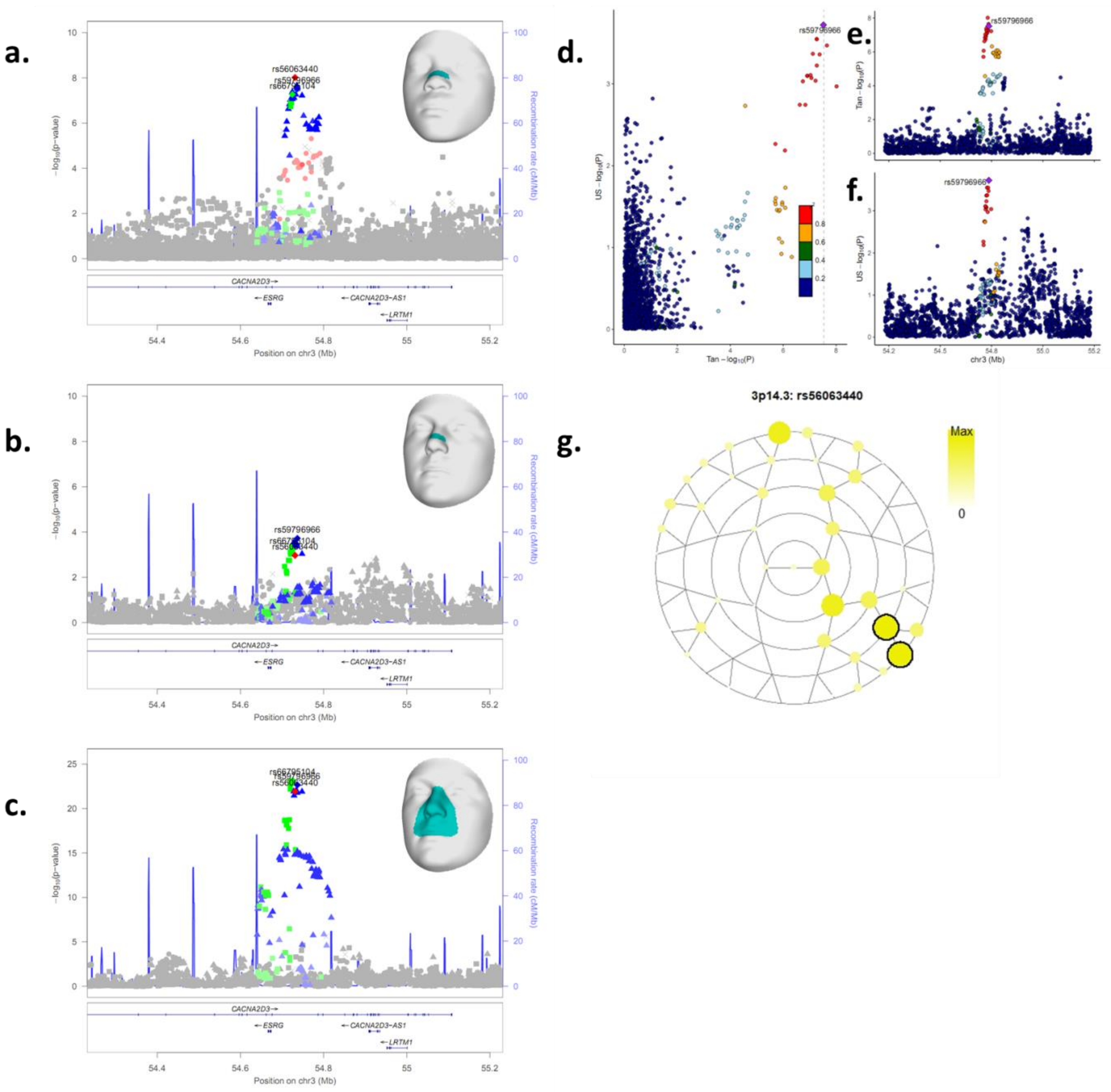

GWAS signal at 3q21.3

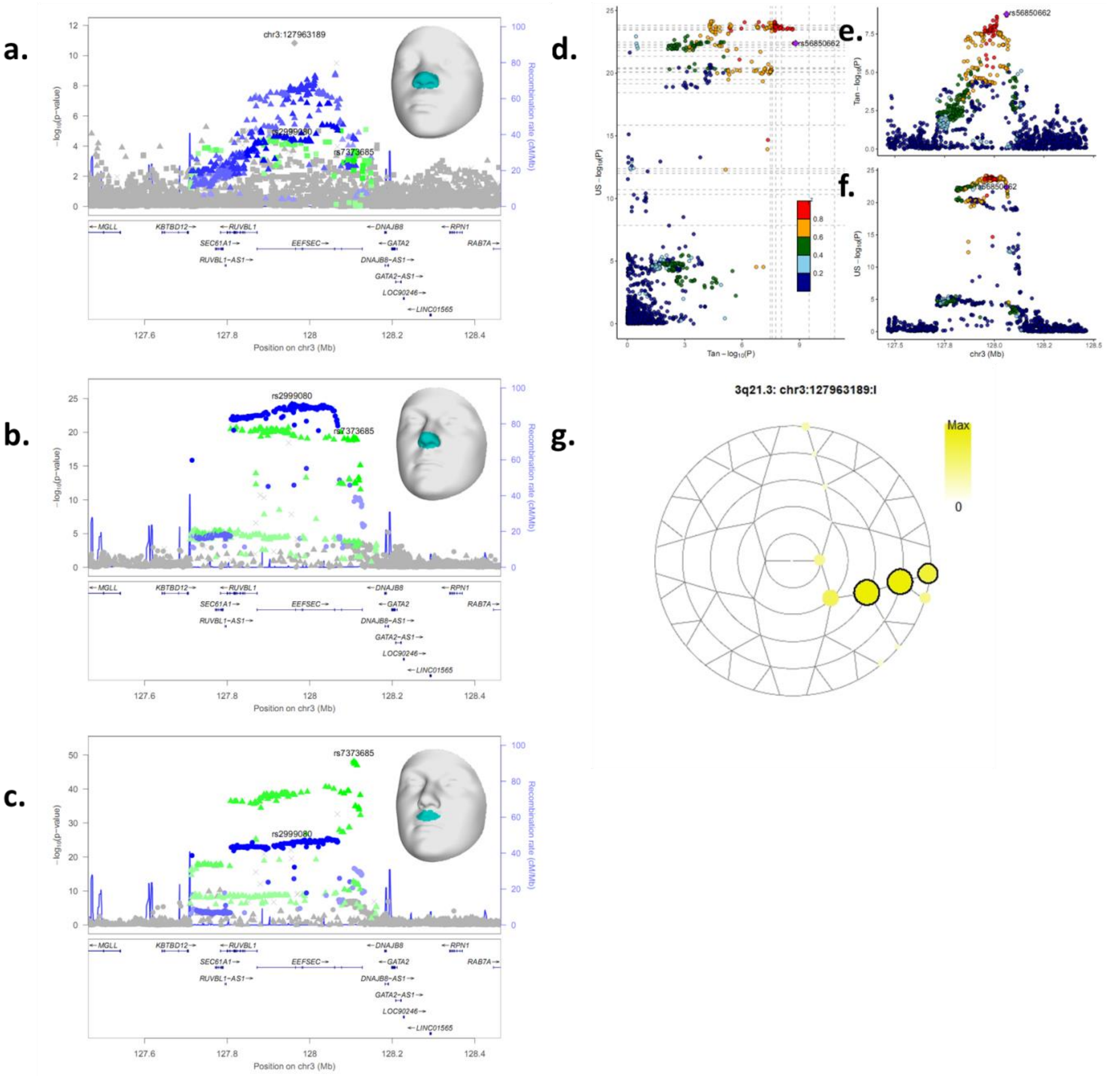

GWAS signal at 3q28

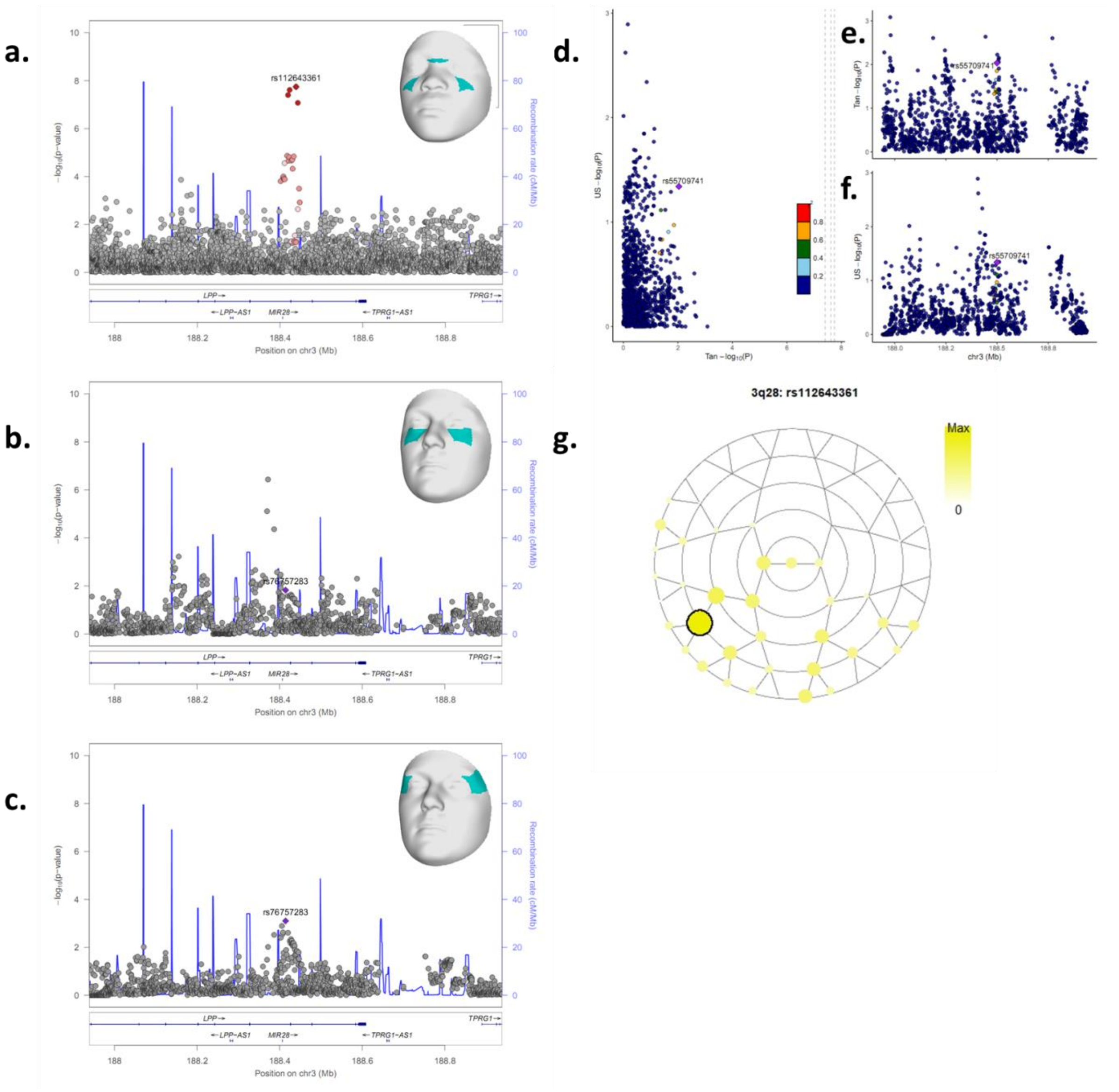

GWAS signal at 4p15.2

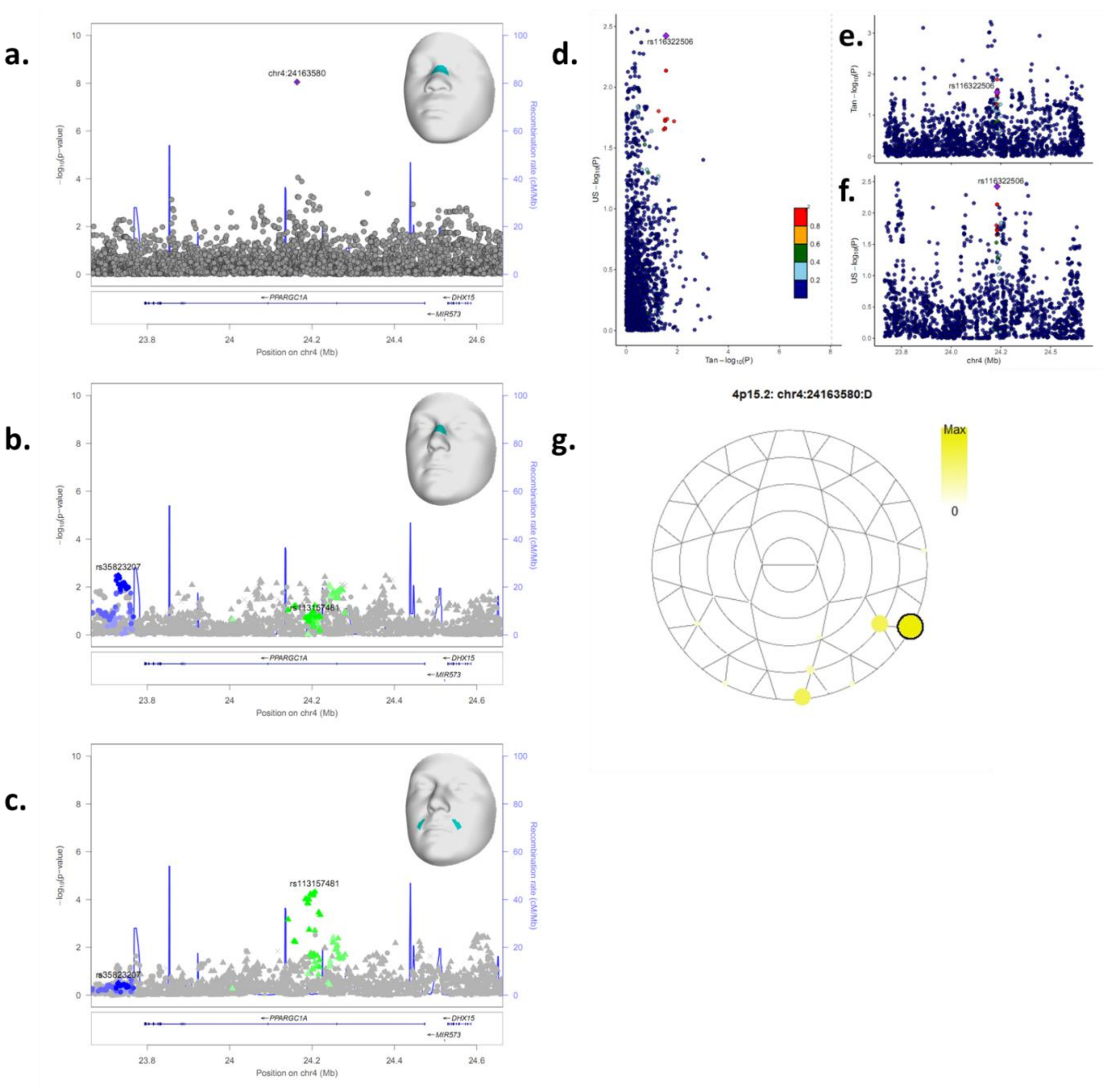

GWAS signal at 4q31.3

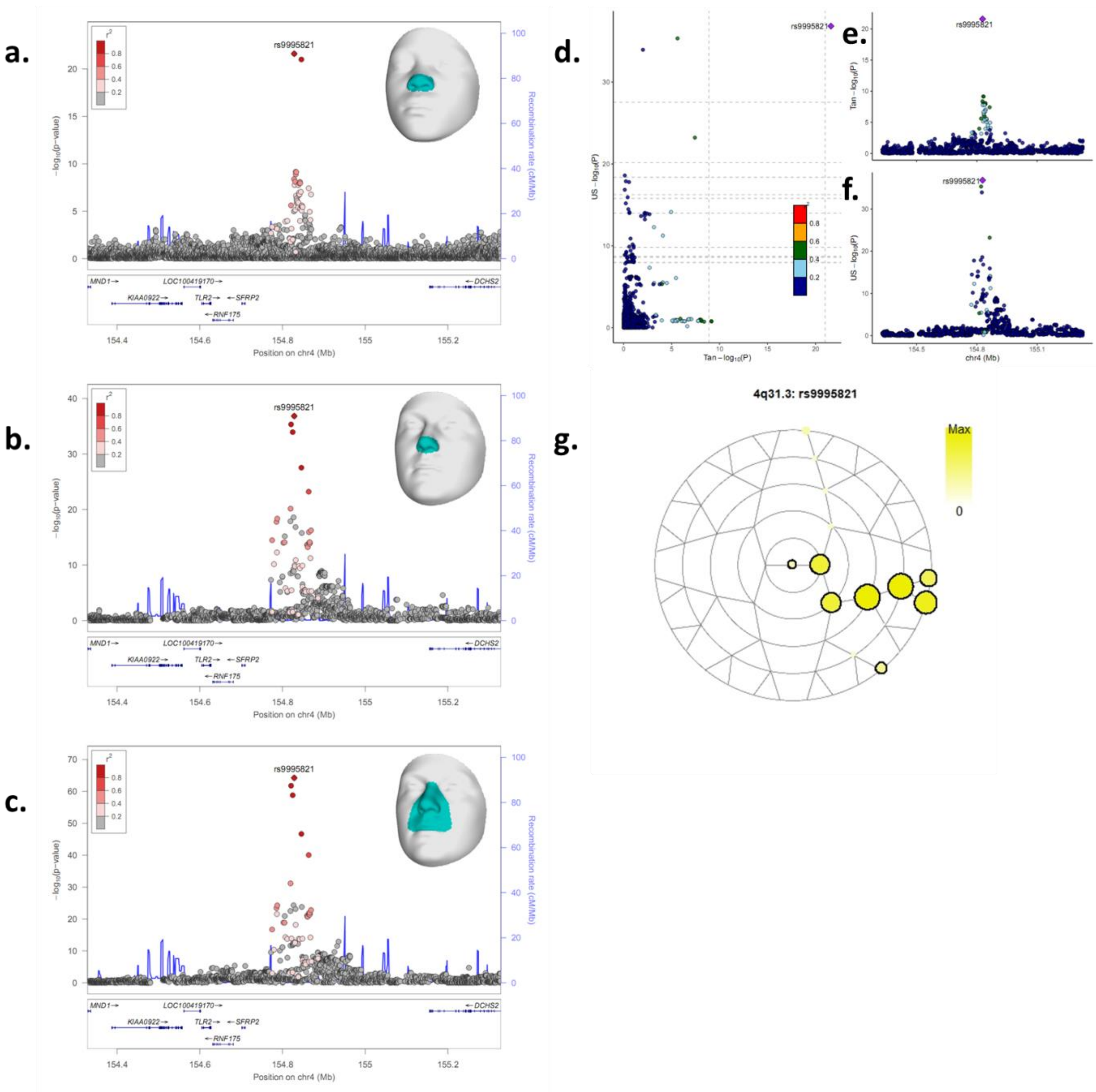

GWAS signal at 5q14.3

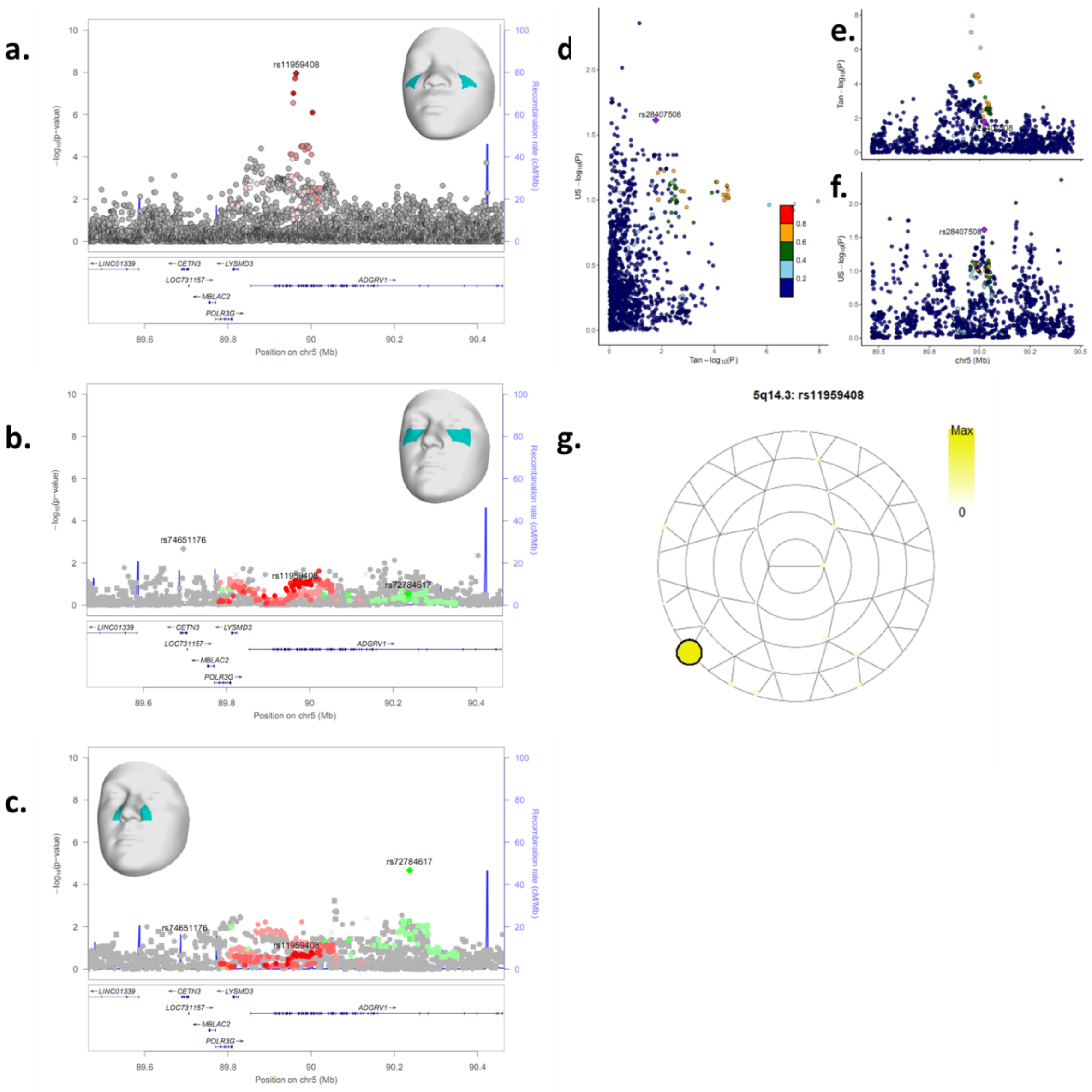

GWAS signal at 5q31.1

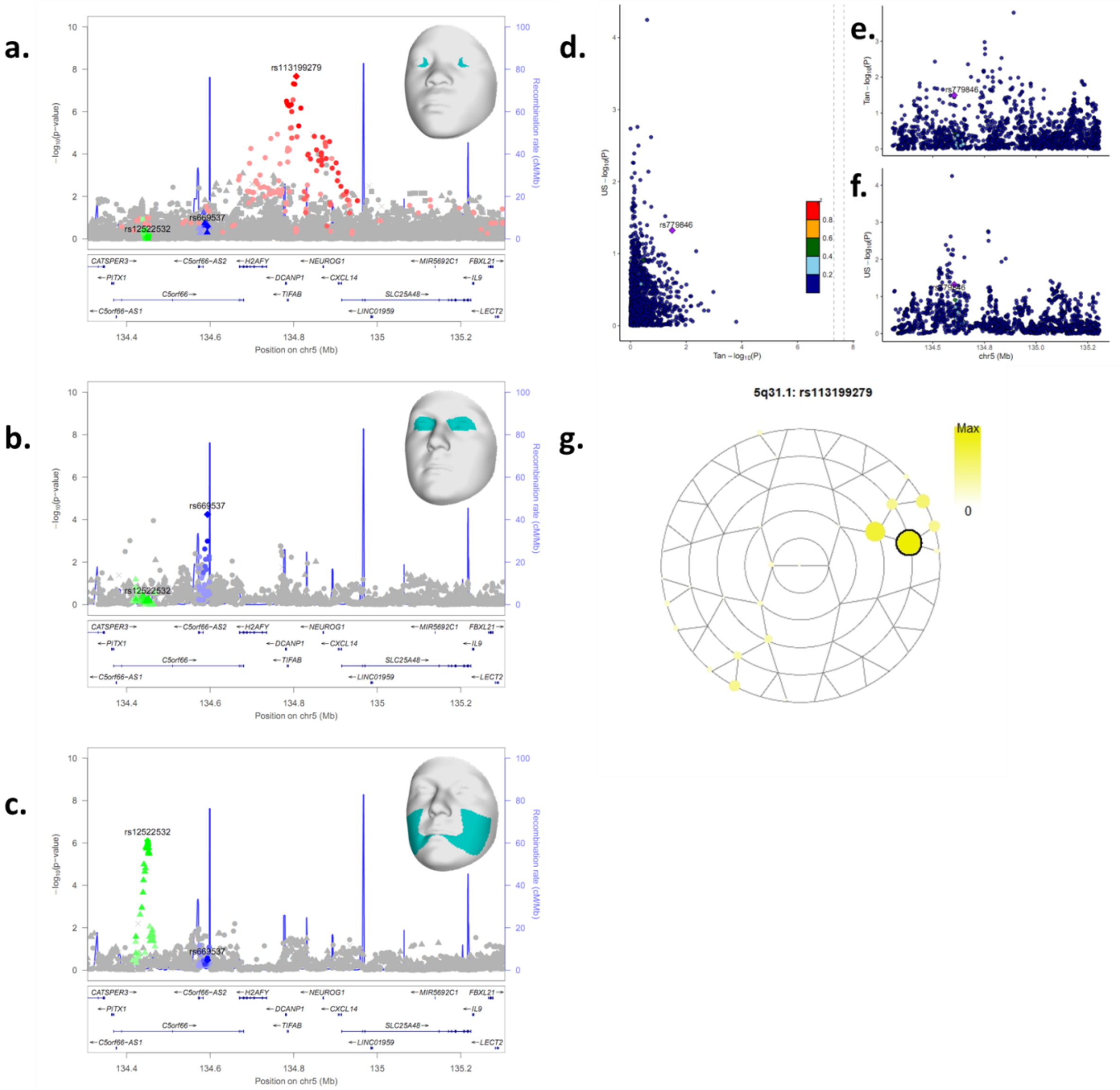

GWAS signal at 7q22.1

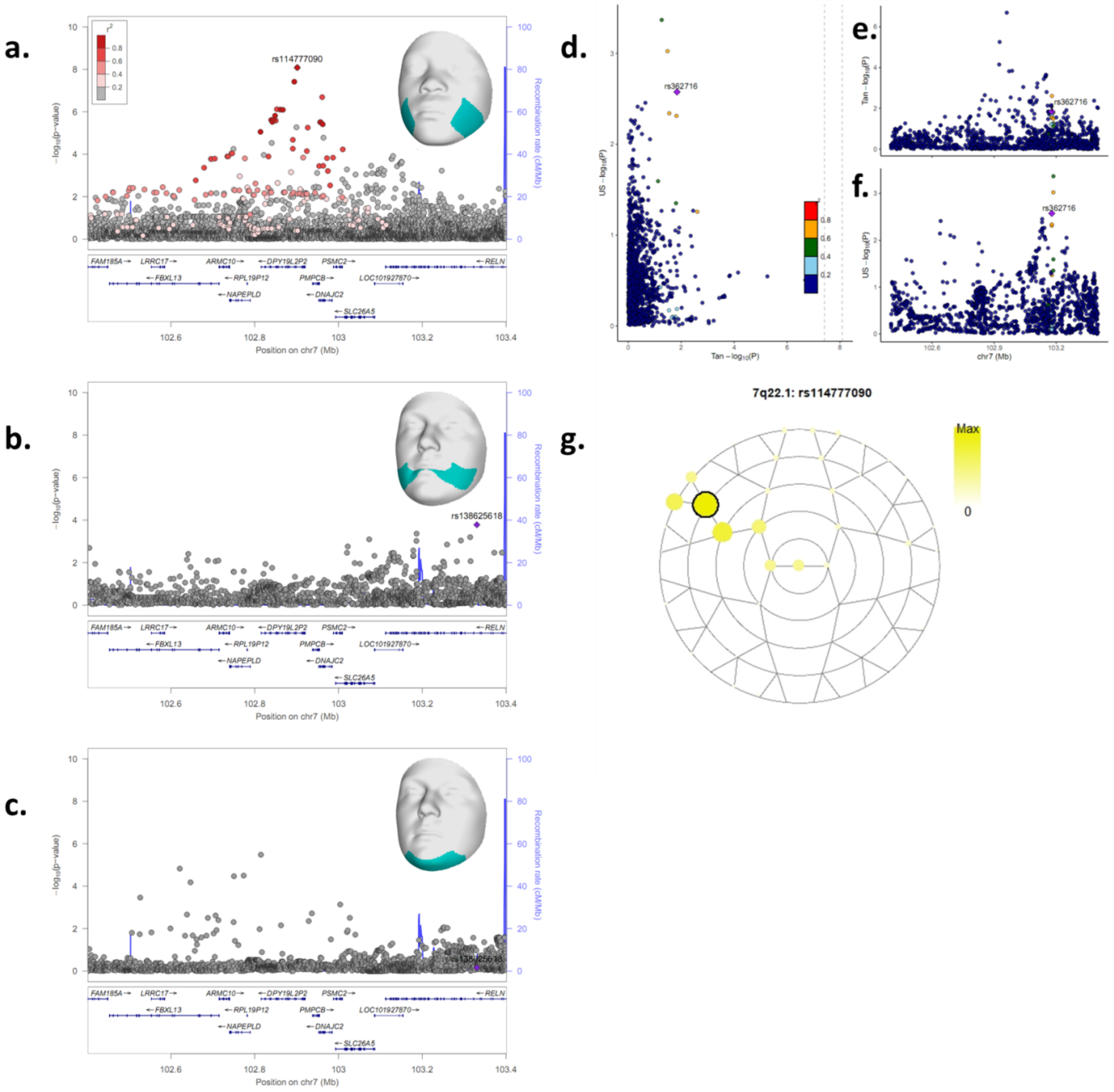

GWAS signal at 9p21.3

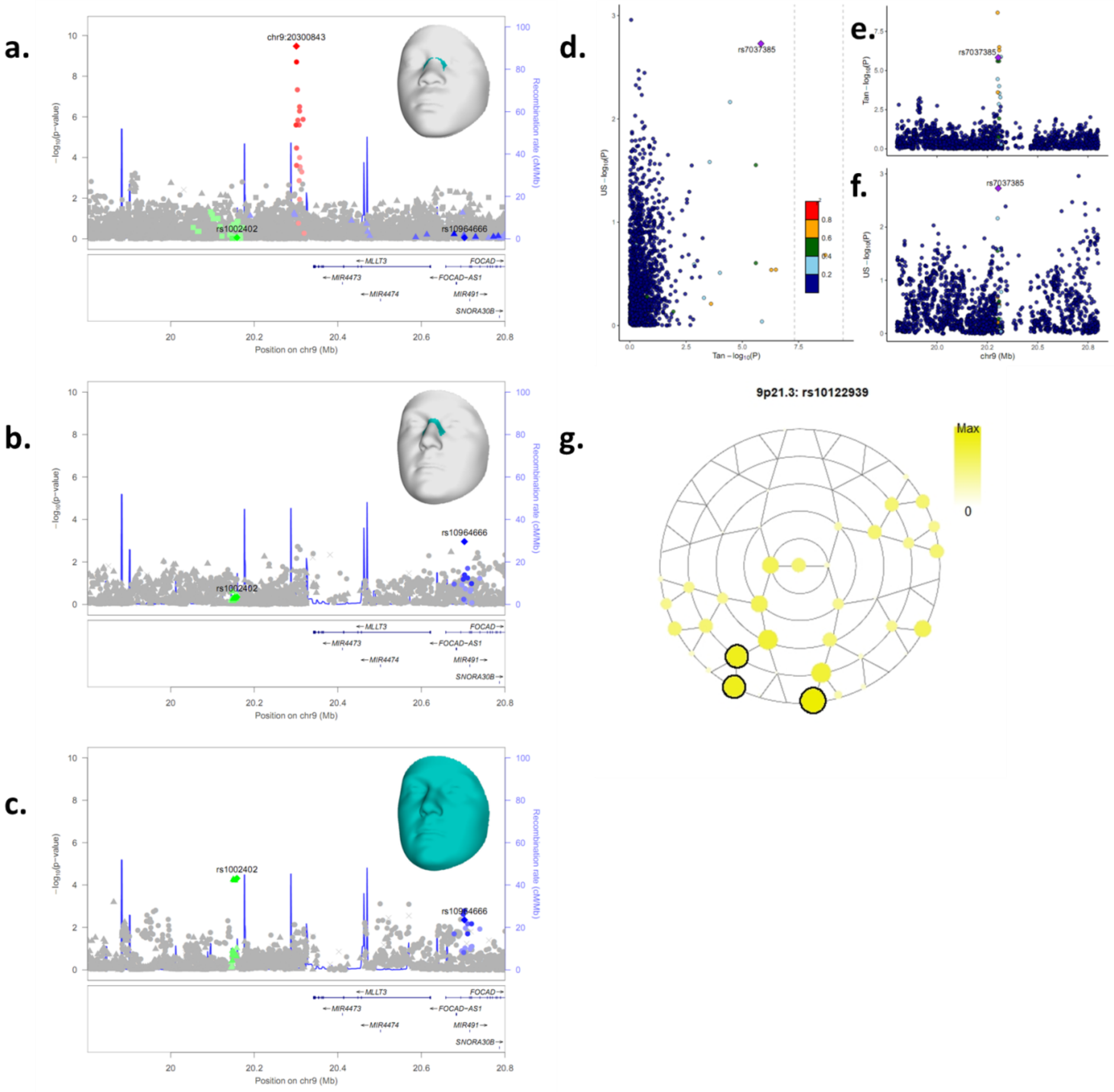

GWAS signal at 9q21.33

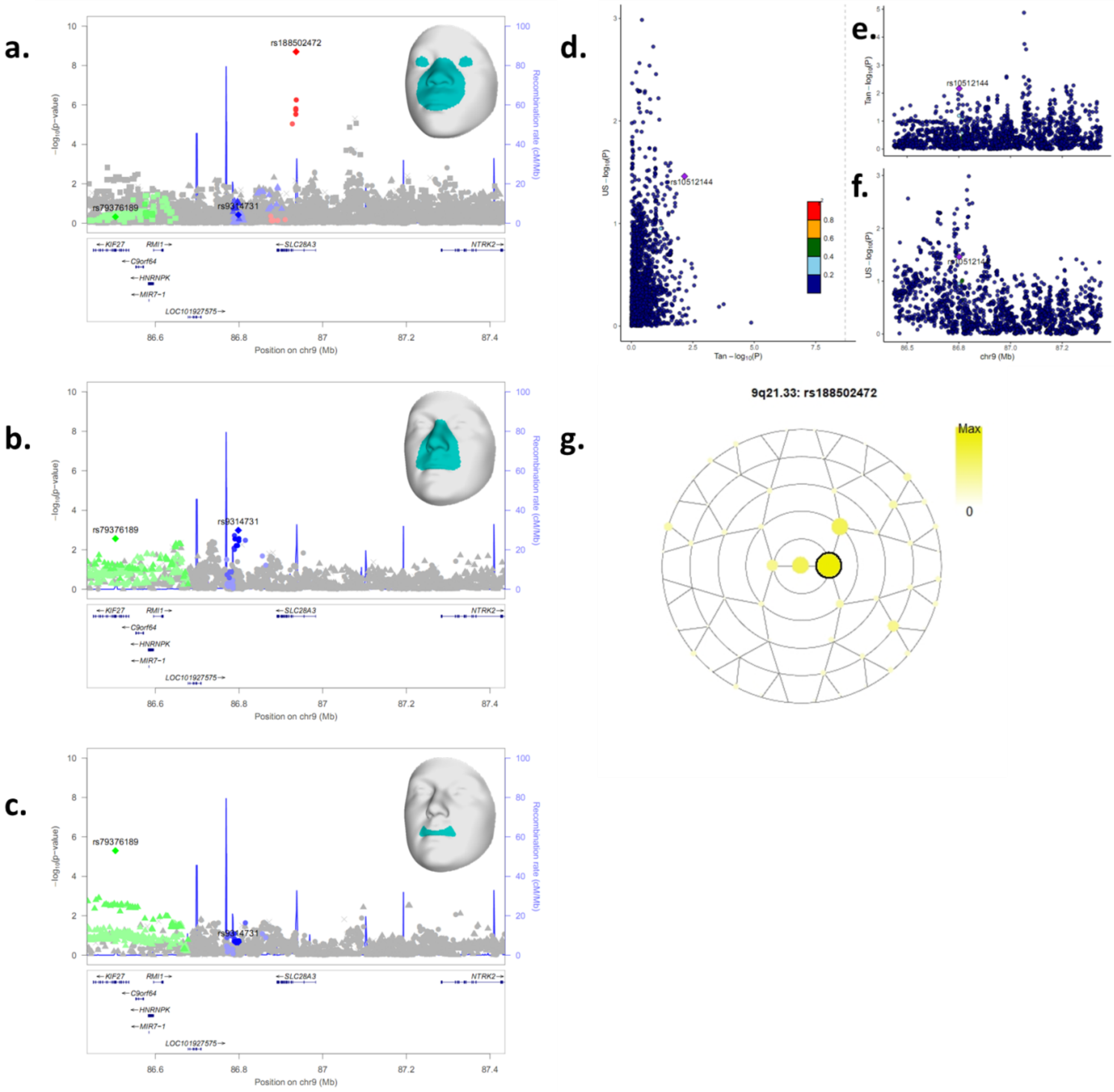

GWAS signal at 10p15.3

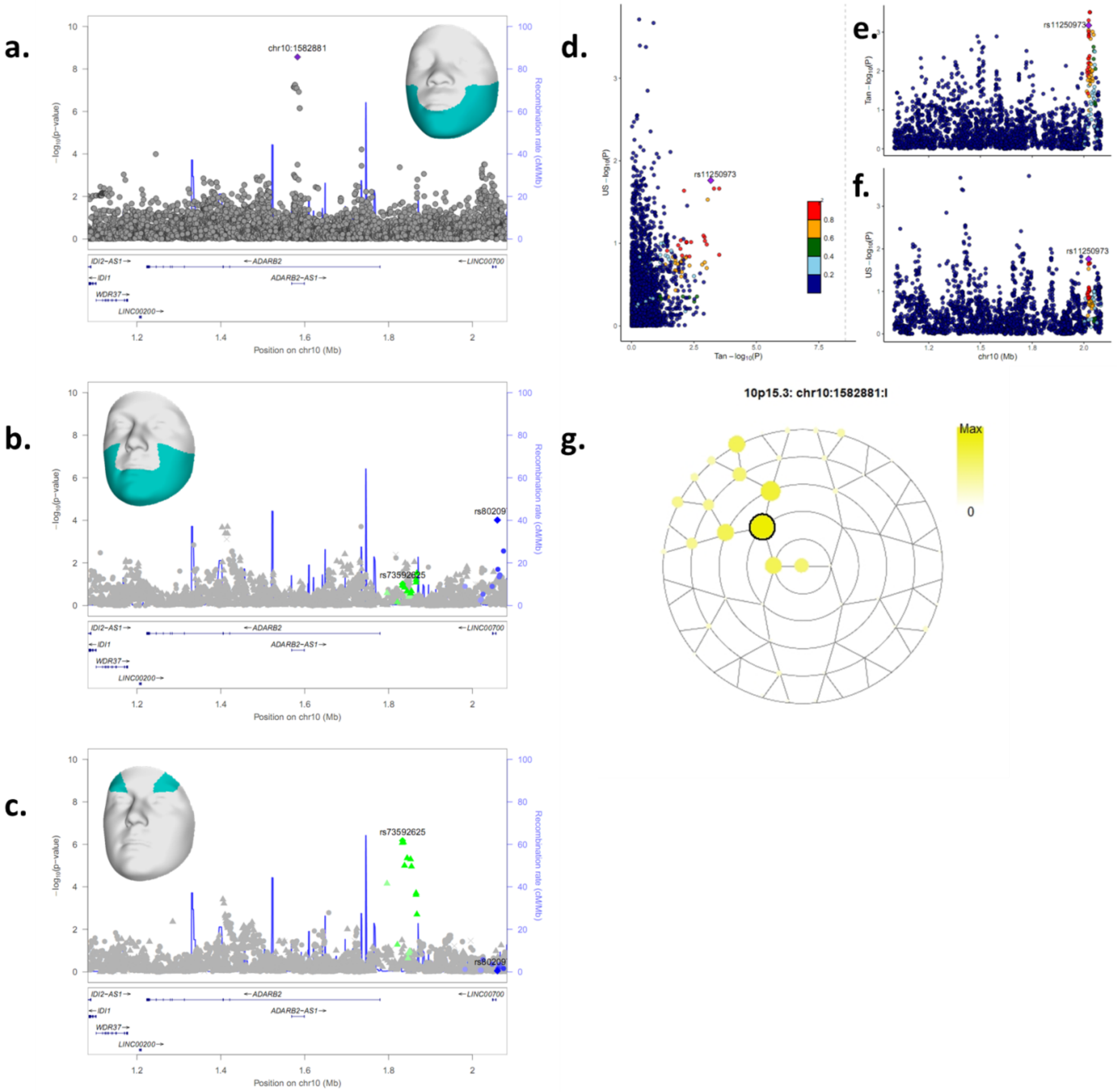

GWAS signal at 10q26.11

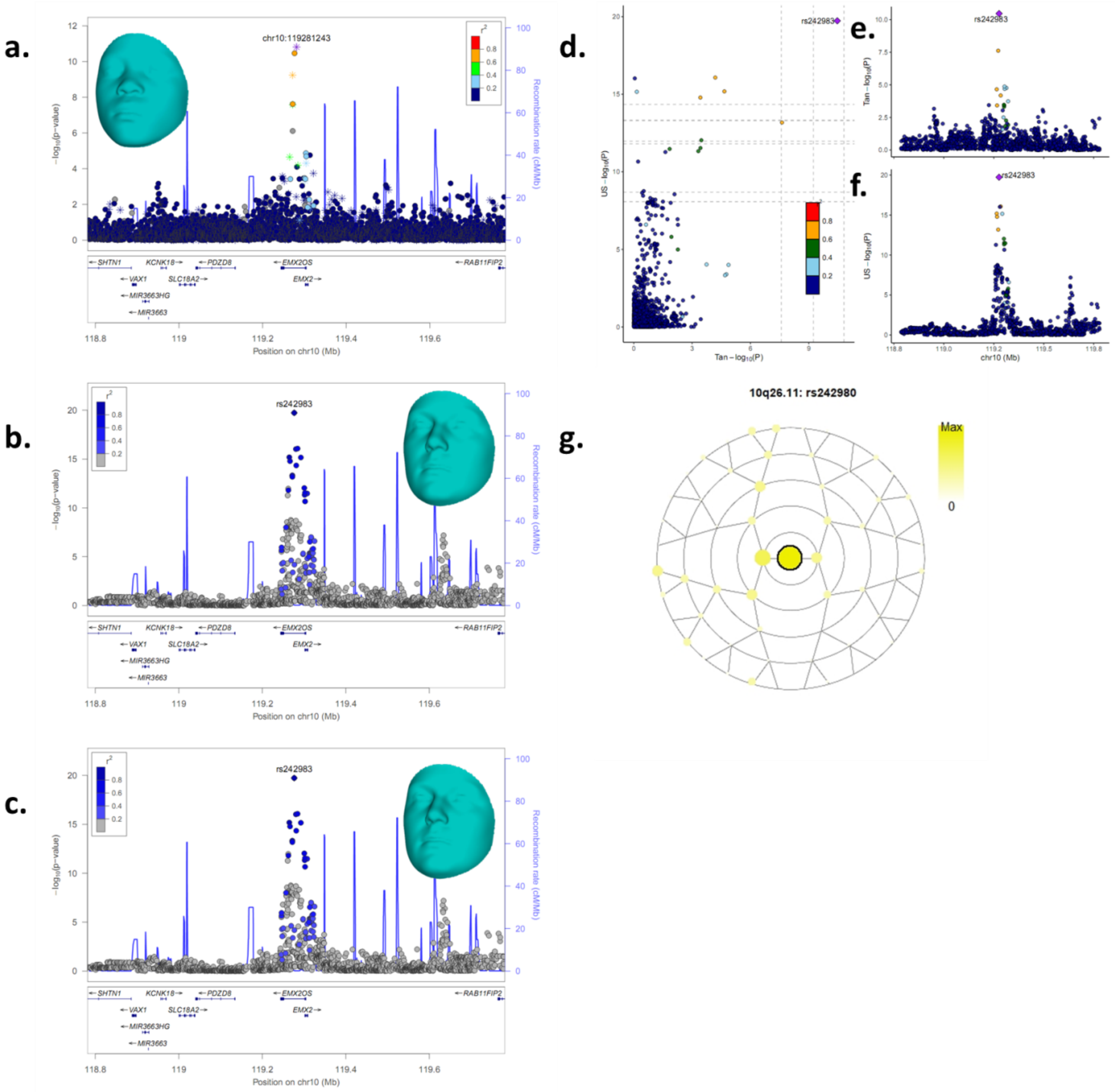

GWAS signal at 12q14.3

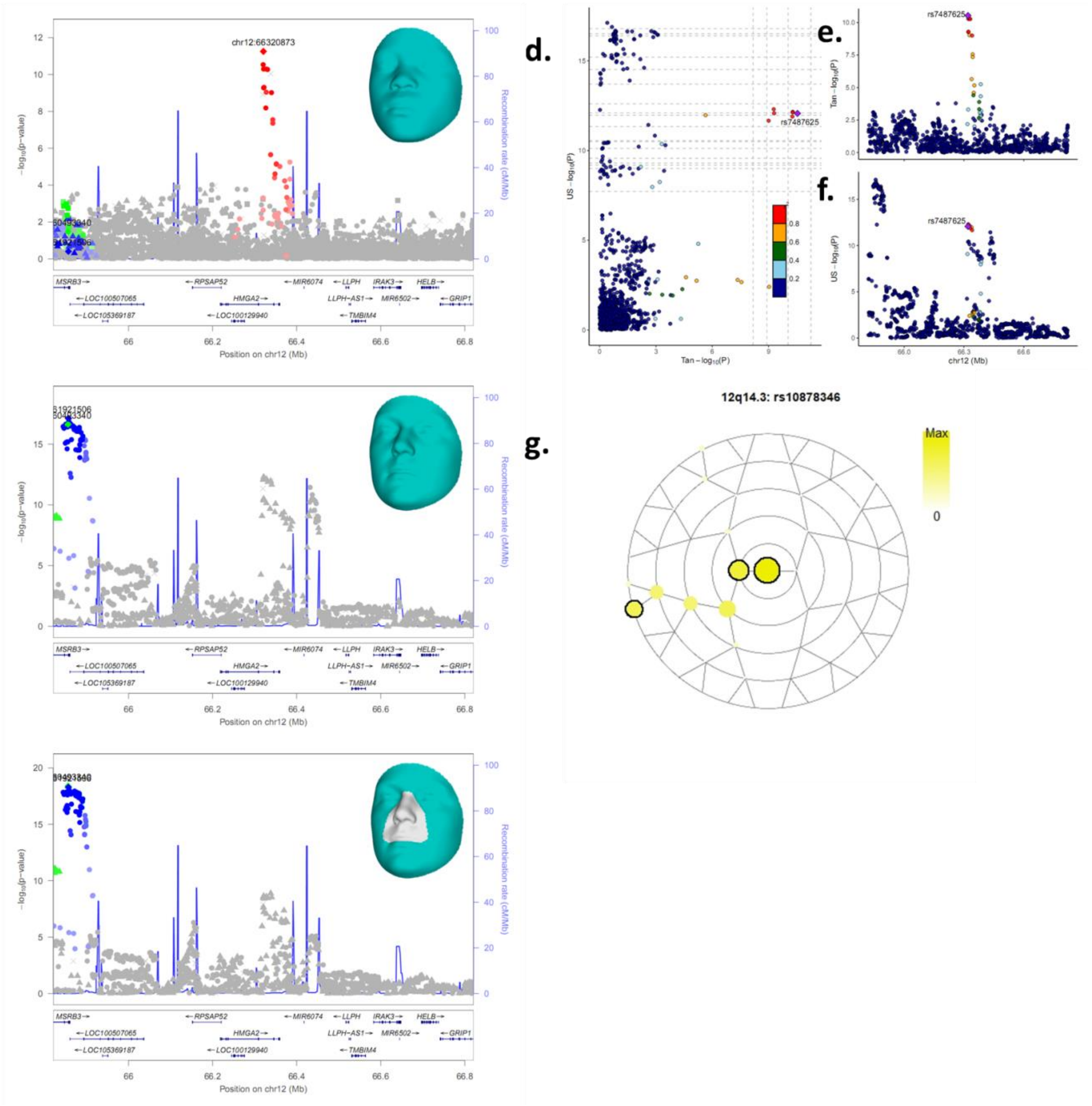

GWAS signal at 12q21.31

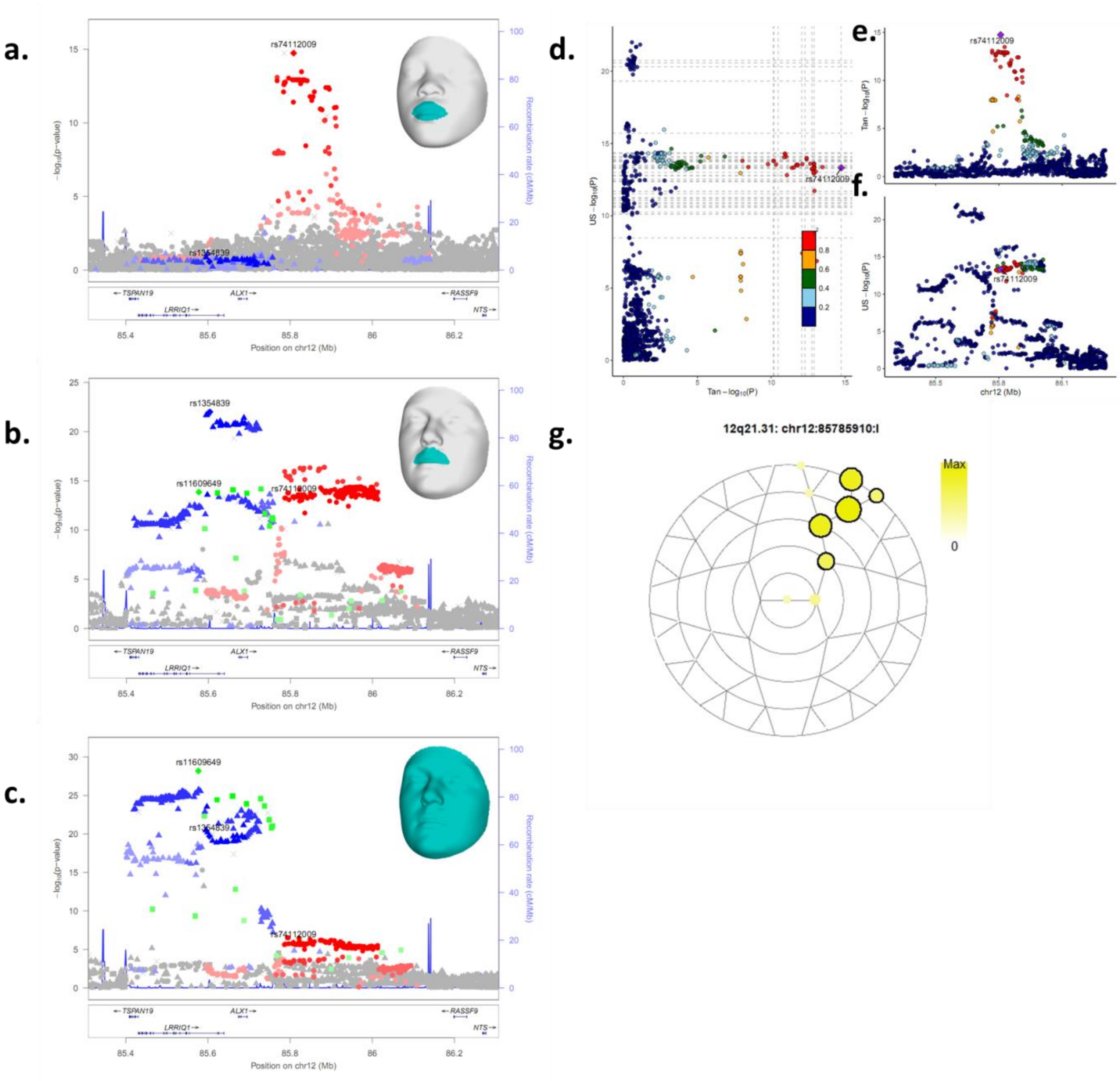

GWAS signal at 12q24.21

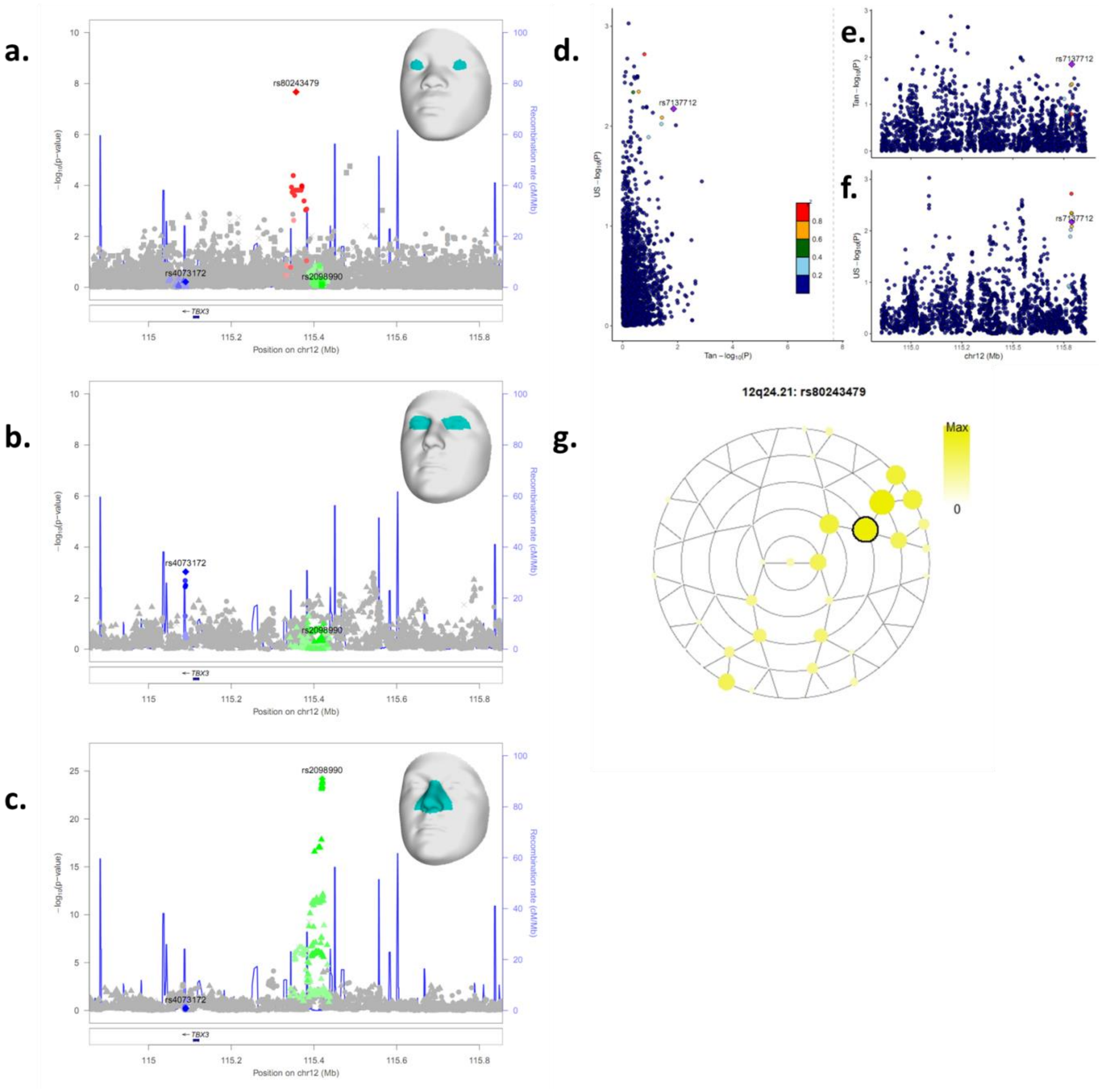

GWAS signal at 13q13.3

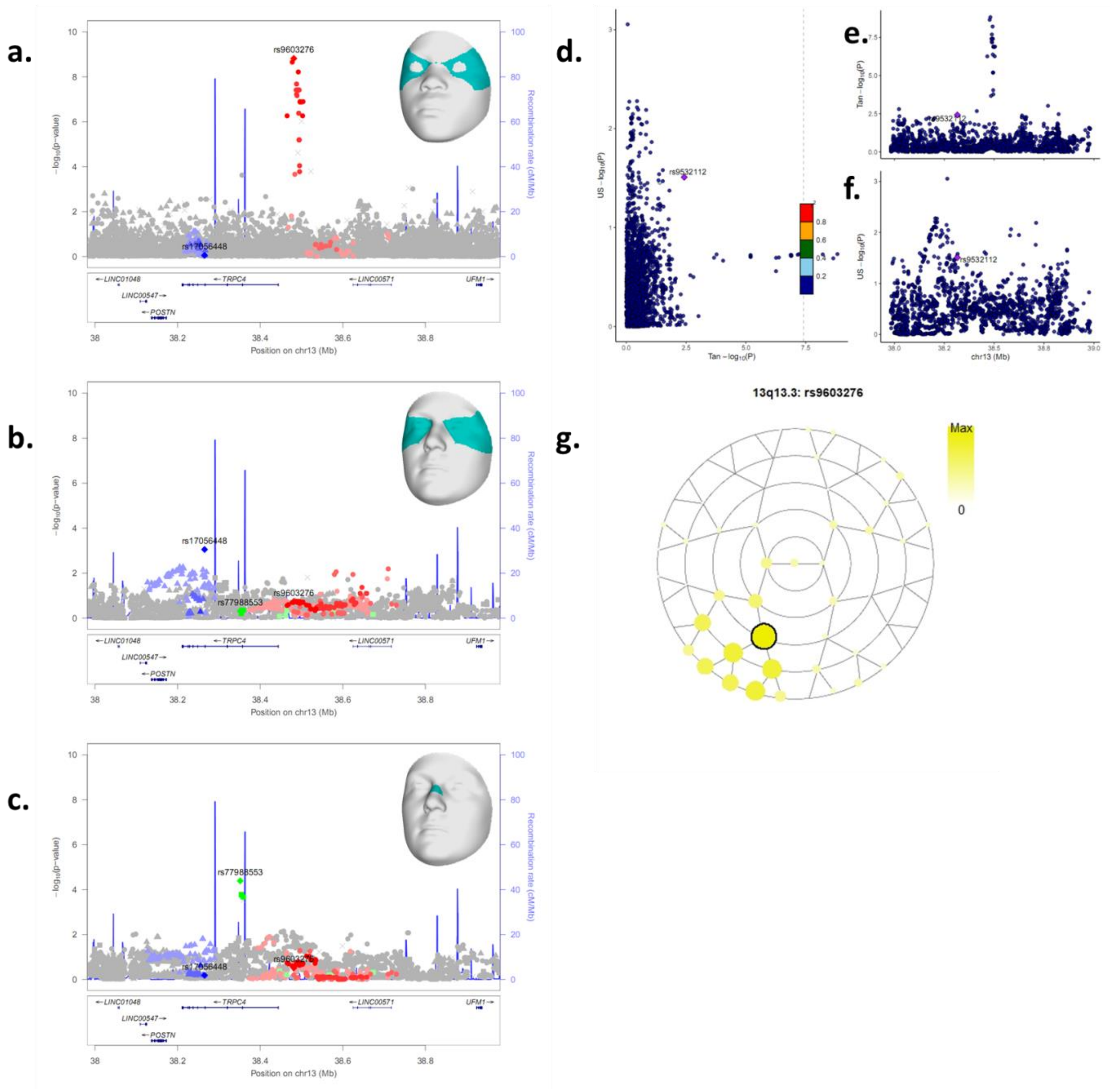

GWAS signal at 13q32.3

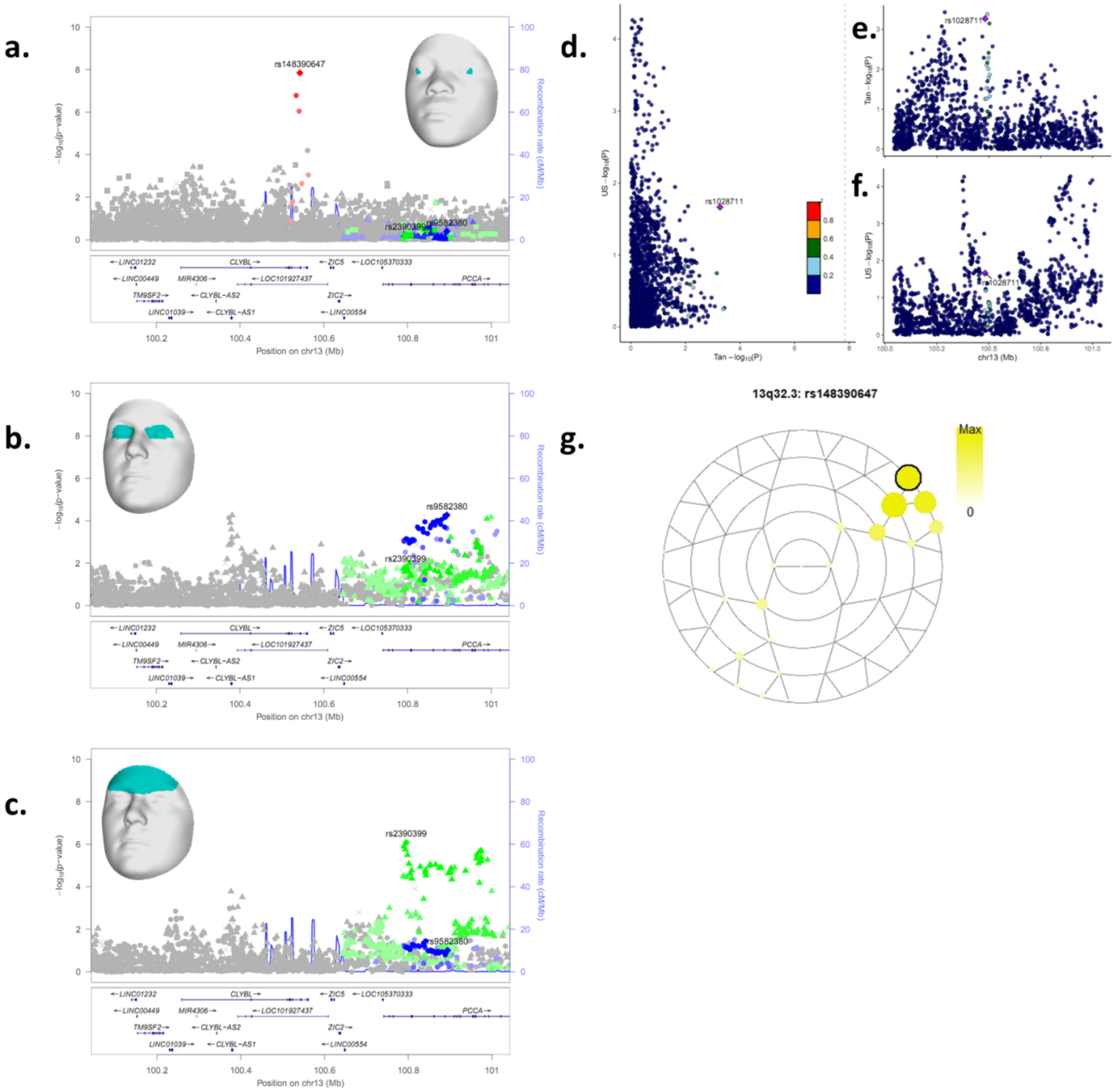

GWAS signal at 18q22.1

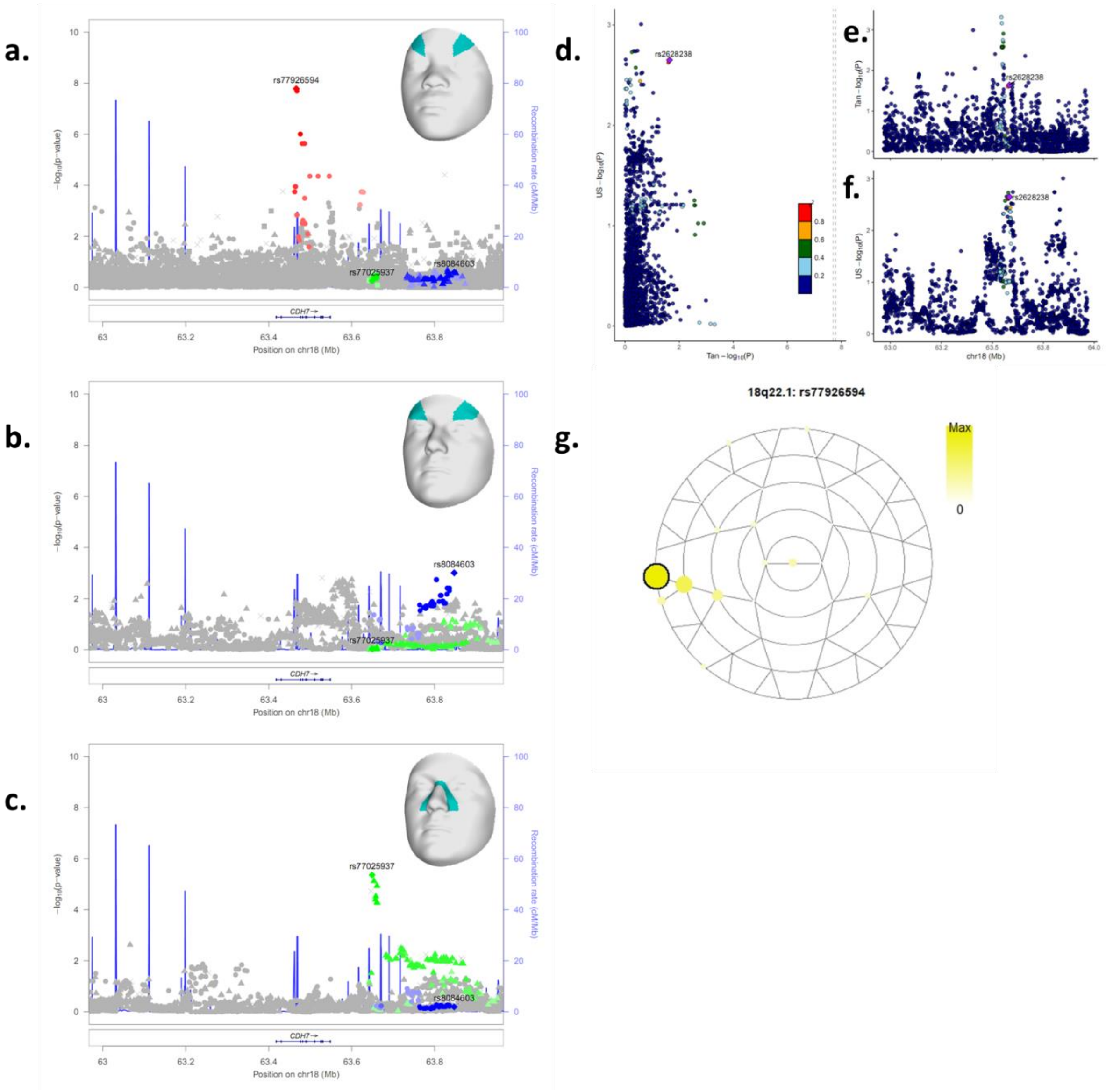

GWAS signal at 20p11.22

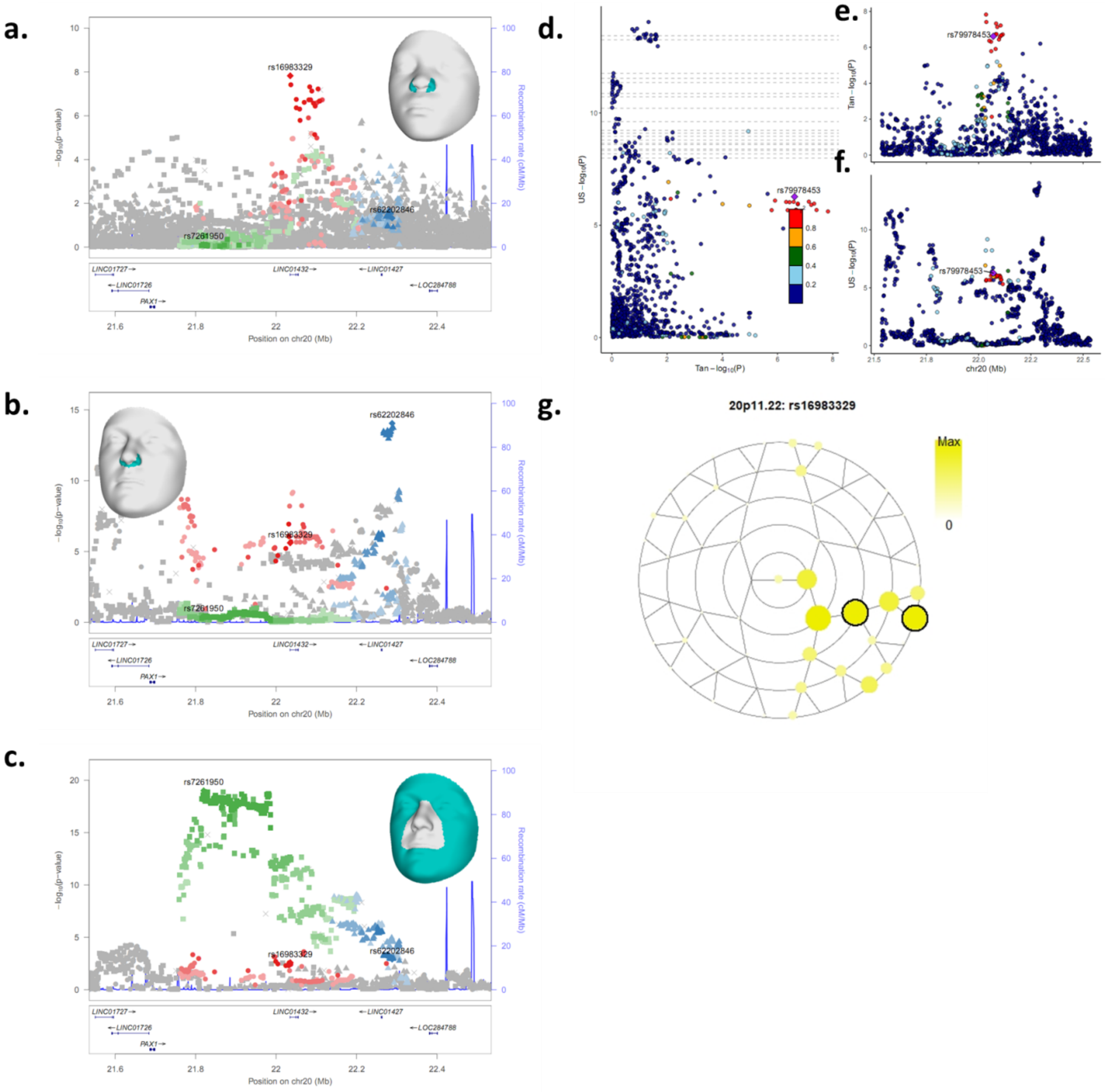

Supplement: S5 Fig — Three LZ plots represent the genetic associations in (a) the best facial segment in Tanzania, (b) a comparable facial segment in Europeans, and (c) the best facial segment in Europeans. (d) A colocalization plot between the Tanzania GWAS and European GWAS at the chromosome 1q22 locus; (e) the association results in the Tanzania GWAS; (f) the association results in the European GWAS. (d-f) The SNP indicated by the purple diamond is the SNP for which the LD information is shown, with African LD structure indicated in the colocalization plot. The vertical gray dashed lines indicate the p-values of SNPs from the Tanzania GWAS that were unavailable in the European GWAS; the horizontal gray dashed lines indicate the p-values of SNPs from the European GWAS that were unavailable in in the Tanzanian GWAS (g) A polar dendrogram showing the global-to-local effect in Tanzania GWAS. Facial segments with a p-value lower than the genome-wide threshold (p = 2.5 × 10^−8^) are circled in black. (PDF) [file pgen.1009695.s005.pdf]
