# Supplementary material for: Genome scans of facial features in East Africans and cross-population comparisons reveal novel associations
Source: PLoS Genet. 2021 Aug 19;17(8):e1009695. doi: 10.1371/journal.pgen.1009695 (PMC8375984; doi:10.1371/journal.pgen.1009695)
Supplement: S1 Appendix — (PDF) [file pgen.1009695.s013.pdf]

Genome Scans of Facial Features in East Africans and Cross-Population Comparisons Reveal Novel Associations

Chenxing Liu[1], Myoung Keun Lee[2], Sahin Naqvi[3,4], Hanne Hoskens[5, 6], Dongjing Liu[1], Julie D. White[7], Karlijne Indencleef[5,8], Harold Matthews[5,6,9], Ryan J. Eller[10], Jiarui Li[5,8], Jaaved Mohammed[3,11], Tomek Swigut[3,11], Stephen Richmond[12], Mange Manyama[13], Benedikt Hallgrímsson[14], Richard A. Spritz[15], Eleanor Feingold[1], Mary L. Marazita[1,2], Joanna Wysocka[3,11,16], Susan Walsh[10], Mark D. Shriver[7], Peter Claes[5,6,8,9], Seth M. Weinberg[1,2,17]*, John R. Shaffer[1,2]*

Affiliations:
[1]Department of Human Genetics, University of Pittsburgh, Pittsburgh, Pennsylvania, United States of America
[2]Department of Oral and Craniofacial Sciences, Center for Craniofacial and Dental Genetics, University of Pittsburgh, Pittsburgh, Pennsylvania, United States of America
[3]Department of Chemical and Systems Biology, Stanford University School of Medicine, Stanford, California, United States of America
[4]Department of Genetics, Stanford University School of Medicine, Stanford, California, United States of America
[5]Medical Imaging Research Center, Katholieke Universiteit Leuven, Leuven, Belgium
[6]Department of Human Genetics, Katholieke Universiteit Leuven, Leuven, Belgium
[7]Department of Anthropology, Pennsylvania State University, State College, Pennsylvania, United States of America
[8]Processing Speech & Images, Department of Electrical Engineering, Katholieke Universiteit Leuven, Leuven, Belgium
[9]Murdoch Children's Research Institute, Melbourne, Victoria, Australia
[10]Department of Biology, Indiana University Purdue University Indianapolis, Indianapolis, Indiana, United States of America
[11]Department of Developmental Biology, Stanford University School of Medicine, Stanford, California, United States of America
[12]Applied Clinical Research and Public Health, School of Dentistry, Cardiff University, Cardiff, United Kingdom
[13]Anatomy in Radiology, Weill Cornell Medicine-Qatar, Doha, Qatar
[14]Department of Anatomy and Cell Biology, Alberta Children´s Hospital Research Institute, University of Calgary, Calgary, Canada
[15]Human Medical Genetics and Genomics Program, University of Colorado School of Medicine, Aurora, Colorado, United States of America
[16]Howard Hughes Medical Institute, Stanford University School of Medicine, Stanford, California, United States of America
[17]Department of Anthropology, University of Pittsburgh, Pittsburgh, Pennsylvania, United States of America
* john.r.shaffer@pitt.edu (JRS); * smwst46@pitt.edu (SMW)

S1 Appendix

The participants in the Tanzania cohort were not required to keep their eyes in the open position during the collection of 3D facial image. To understand the effect of this limitation of the data collection on our study we performed a sensitivity analysis as described in this Appendix.

Unfortunately, we did not have access to any data on eye position (open vs. closed) other than what is available in the publicly released 3D facial images, so we can only determine open vs. closed position from the 3D images themselves. The 3D facial shape images, which are devoid of coloration and texture, cannot always be unambiguously scored by a human rater as to whether the eyes are open or closed. To illustrate this, below are a few example faces where the eyes appear to be closed (left), open (center), or undetermined based on visual inspection by a human rater.

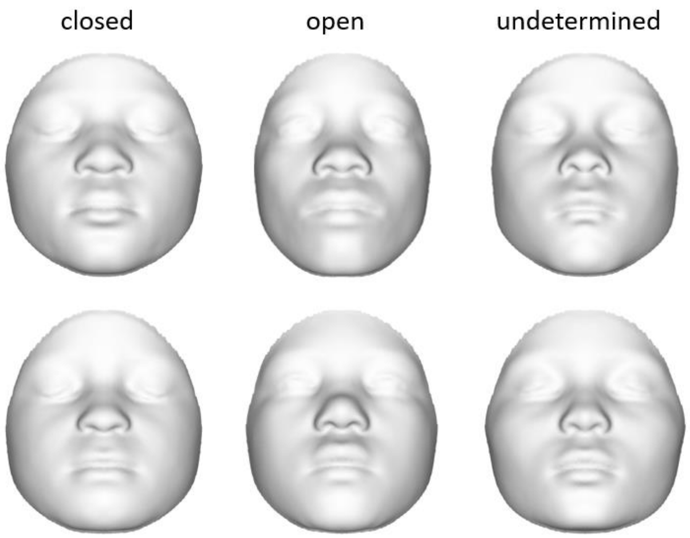

To more deeply explore this issue, we performed an approach that involved manually scoring facial images by a human rater to train a machine learning classifier. In brief, we manually scored the eyes of 1000 facial images and determined that 377 (37.7%) were open, 260 (26%) were closed, and 363 (36.3%) could not be determined. We then built a classification model via linear support vector machines using the 637 human-rated open or closed eye images as input. Five-fold cross-validation showed this classifier had accuracy of approximately 98% (defined as concordance with eye positions scored by the human rater, which may not reflect the true state 100% of the time). Next, we applied this classifier to the entire dataset of 2,595 3D facial images. The distribution of the posterior probability of open eyes for the sample is provided, below.

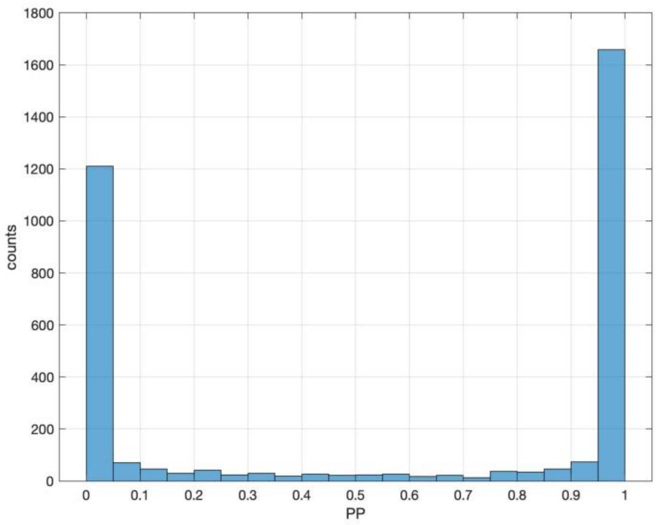

The distribution of the posterior probability of open eyes was bimodal with a majority of images having very high or very low probability. We assigned images with posterior probability >0.5 as open, and <0.5 as closed. Based on this threshold, 56% of participants had imaging with open eyes, and 44% of participants had imaging with closed eyes.

We next explored what effect the incorporation of information about predicted eye position would have on our analyses. We included the "eyes open" or "eyes closed" states as an additional covariate to adjust for in our facial shape model. Eye state explained 4.2% of the residual facial variation (after adjustment for other covariates) and, as expected, had the largest effect in the eye region. This can be seen in the top row of the figure below, where the face model with the closed eye (left) and open eye (middle) states are depicted, as well as a heatmap (right) showing the effect of eye state on the face. After adjustment for open vs. closed eye state (bottom row), the eye state covariate explains essentially 0% of the residual facial variation, the face model for both closed and open eyes are indistinguishable, and the heatmap shows no lingering effect across the face, which all indicate that variation due to eye state was adequately captured in the model.

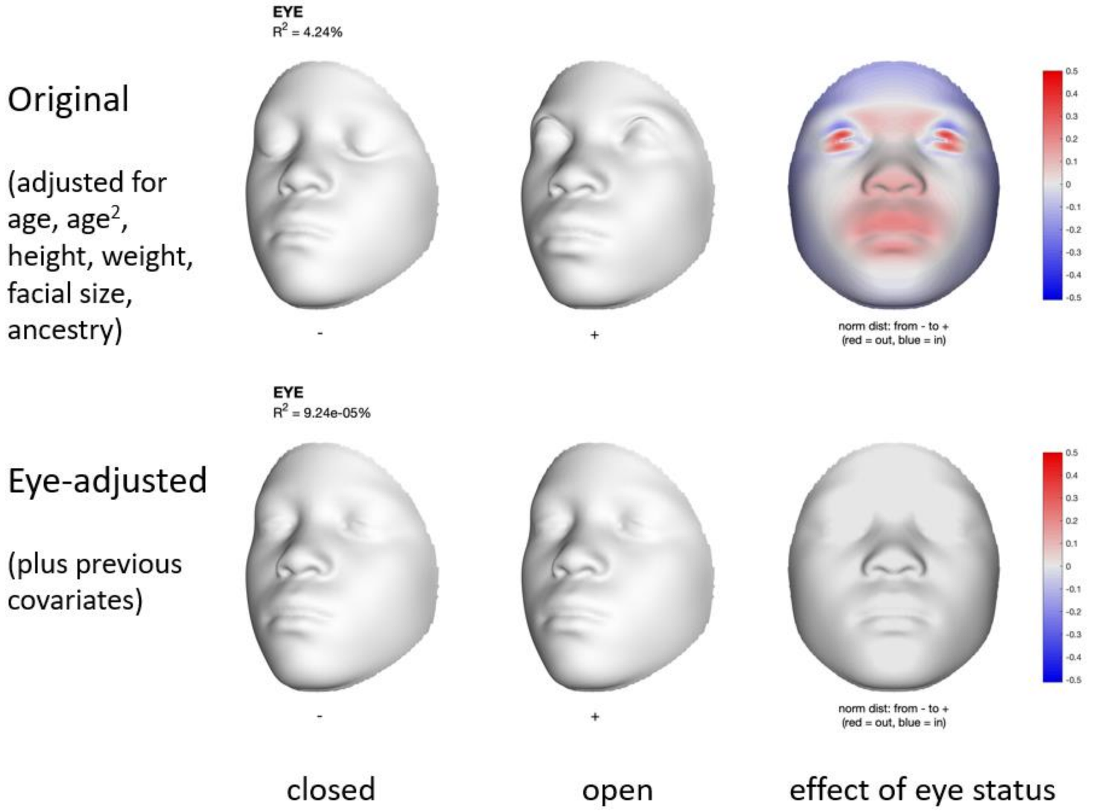

Next, we repeated the segmentation of the face into modules and generated new multi-dimensional facial shape phenotypes. The segmentation was quite similar to the original, with the exception of the eye region. Indeed, across the 6 levels of the hierarchy, the overlap in modules defined as the normalized mutual information was quite high: 100% for module 1, 80.7% for modules 2-3, 87.6% for modules 4-7, 84.3% for modules 8-15, 82.8% for modules 16-31, and 80.0% for modules 32-63. The eye-adjusted and original segmentations are shown below (note, the arbitrary flip in positions in the rosette of the blue “nose” and green “lips” quadrants).

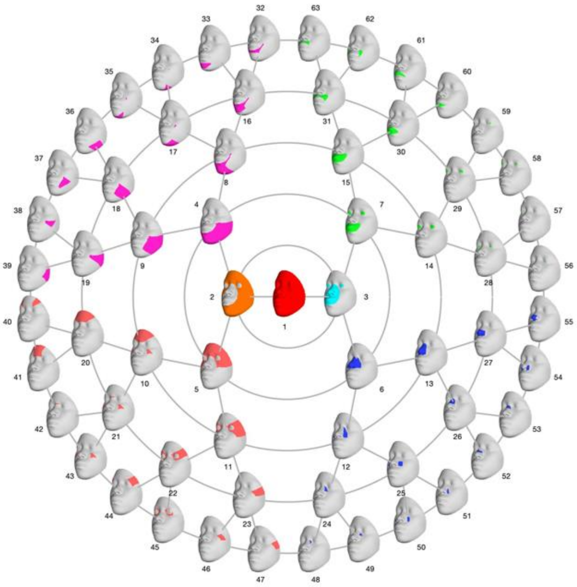

original segmentation

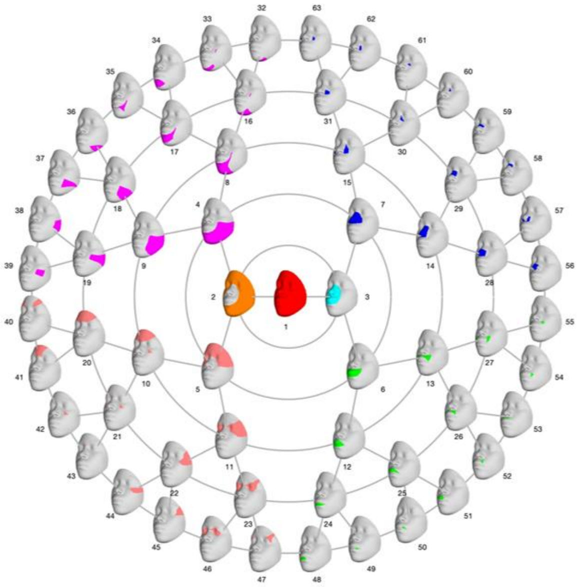

updated segmentation
(+ eye adjustment)

We then repeated our genetic analysis on the new phenotypes. Association results for 18 of the 20 top hits were similar, with exact p-values being a bit different. Two of the 20 hits, which were observed for the original modules 28 and 59, went away after adjustment for eye state. This makes sense because the new eye state-adjusted segmentation did not yield segments representing regions of the face comparable to the original modules 28 and 59, which are restricted to the eyes.

Table comparing results for top SNPs between eye-adjusted and original analyses

					original analysis			eye-adjusted analysis	
Lead SNP	Position	Chr	A1	A2	Best P	Best module	No. modules	Best P	Best module
rs58409393	155025307	1	G	A	1.6E-08	41	1	4.4E-09	41
rs56063440	54731374	3	C	G	9.7E-09	52	3	6.7E-08	59
chr3:127963189	127963189	3	T	TGC	1.5E-11	27	3	1.4E-11	28
rs112643361	188438871	3	G	A	1.8E-08	21	1	1.0E-08	21
chr4:24163580	24163580	4	G	GAT	8.9E-09	53	1	2.0E-08	58
rs9995821	154828366	4	C	T	2.5E-22	27	8	1.7E-21	28
rs11959408	89964298	5	T	C	1.1E-08	43	1	9.1E-06	42
rs113199279	134806314	5	T	G	2.1E-08	28	1	**1.8E-03**	45
rs114777090	102901689	7	G	A	8.2E-09	18	1	3.7E-08	18
rs10122939	20300843	9	G	A	3.3E-10	48	5	2.0E-08	46
rs188502472	86936444	9	T	C	2.0E-09	3	1	5.3E-06	1
chr10:1582881	1582881	10	AC	A	2.7E-09	4	1	4.2E-09	4
rs242980	119281243	10	A	G	1.5E-11	1	2	1.8E-11	1
rs10878346	66320873	12	A	G	5.5E-12	1	4	4.5E-12	1
rs74112009	85808404	12	A	T	1.8E-15	30	6	1.8E-13	12

rs80243479	115356683	12	C	T	2.1E-08	14	1	4.2E-06	46
rs9603276	38481292	13	G	A	1.5E-09	11	1	4.2E-09	11
rs148390647	100542948	13	G	C	1.4E-08	59	1	**1.1E-04**	5
rs77926594	63466440	18	A	G	1.6E-08	40	1	2.2E-07	40
rs16983329	22035197	20	A	G	1.5E-08	54	2	7.0E-09	57

Chr: chromosome

A1, A2: alleles

Best P: the smallest p-value across the the 63 modules

Best Module: the module number corresponding to the rosette with the smallest p-value

No. Modules: the number of significantly associated modules

Bold: large p-values indicating little evidence of association
